# Demographic effects of interacting species: exploring stable coexistence under increased climatic variability in a semiarid shrub community

**DOI:** 10.1038/s41598-021-82571-z

**Published:** 2021-02-04

**Authors:** Ana I. García-Cervigón, Pedro F. Quintana-Ascencio, Adrián Escudero, Merari E. Ferrer-Cervantes, Ana M. Sánchez, José M. Iriondo, José Miguel Olano

**Affiliations:** 1 Biodiversity and Conservation Area, Rey Juan Carlos University, 28933 Móstoles, Spain; 2 Department of Biology, University of Central Florida, Orlando, FL 32816 USA; 3 iuFOR-EiFAB, University of Valladolid, Campus Duques de Soria, 42004 Soria, Spain

**Keywords:** Ecology, Climate-change ecology, Community ecology, Population dynamics

## Abstract

Population persistence is strongly determined by climatic variability. Changes in the patterns of climatic events linked to global warming may alter population dynamics, but their effects may be strongly modulated by biotic interactions. Plant populations interact with each other in such a way that responses to climate of a single population may impact the dynamics of the whole community. In this study, we assess how climate variability affects persistence and coexistence of two dominant plant species in a semiarid shrub community on gypsum soils. We use 9 years of demographic data to parameterize demographic models and to simulate population dynamics under different climatic and ecological scenarios. We observe that populations of both coexisting species may respond to common climatic fluctuations both similarly and in idiosyncratic ways, depending on the yearly combination of climatic factors. Biotic interactions (both within and among species) modulate some of their vital rates, but their effects on population dynamics highly depend on climatic fluctuations. Our results indicate that increased levels of climatic variability may alter interspecific relationships. These alterations might potentially affect species coexistence, disrupting competitive hierarchies and ultimately leading to abrupt changes in community composition.

## Introduction

Increased climate variability has been identified as one of the major challenges to population persistence. Changes in normal patterns of climatic events affect organisms’ vital rates, altering their population dynamics and threatening population persistence^1,2^. In natural communities, populations are not isolated; they interact with each other in such a way that climate effects on a single-species population may ultimately impact the dynamics of the whole community. The stochastic nature of climatic variations poses an additional challenge to understand their demographic effects^3,4^, particularly when these variations drive differential responses in populations of coexisting species. The existence of idiosyncratic responses to common climatic fluctuations acts as a mechanism limiting competition and thus favouring species coexistence in the long term^5–7^. Obviously, an alternative scenario in which responses to climate are similar among coexisting species is also plausible and would indicate, for instance, that species coexistence is promoted by other factors such as fine-scale environmental heterogeneity driven by soil characteristics, species composition or grazing^8–10^ and by niche differences^11^.

Despite the undisputed importance of climate variability for population dynamics, its effects may, in fact, be strongly modulated by biotic interactions^12,13^. Plants modify their fine-scale environment altering physical conditions for other plants, improving or reducing their access to resources^14,15^. For instance, moist microhabitats and favourable years in water-limited systems may result in weakened plant–plant interactions, while years with water scarcity can magnify species competition for this limiting resource^16^. Integrating the effects of plant–plant interactions on demographic models is, however, challenging: different vital rates may be simultaneously affected by biotic interactions, resulting in contrasting outcomes that may either cancel each other out or act synergistically on population dynamics^17,18^. In addition, the intensity and direction (positive or negative) of plant–plant interactions shift over time due to ontogeny^19^. As a consequence, large individuals can serve as nurses for seedlings of conspecific or heterospecific plants, outcompeting them when adults^20^.

Current efforts to integrate the consequences of species interactions into demographic models involve different approaches. A first one considers the community as a fixed template in which a target species thrives (e.g.^21–23^). A more sophisticated but realistic perspective considers plant communities as a fluid environment in which populations modify their dynamics depending on climate and on environmental determinants, but also in response to both own and coexisting species’ dynamics^24^. Multispecies demographic models are a promising analytical approach to consider this fluid perspective^25,26^. In particular, the use of integral projection models (IPMs^27,28^) allows summarizing population dynamics as a function of different covariates that impact vital rates^29,30^. Here, we followed this second approach to assess how climate variability affects species persistence and coexistence, using two dominant plant species in a semiarid shrub community on gypsum soils as a case study.

Since water limitation is a primary demographic constraint in semiarid ecosystems, it is expected that the forecasted increase in drought frequency and intensity^31^ will magnify its demographic impacts. Extreme droughts may trigger mortality episodes^32^, but recruitment is also very sensitive to water availability and concentrates in windows with wet conditions, which could boost population growth. These recruitment peaks could act as “rescue” events^33,34^ and leave a long-term imprint in population cohort-structure^35,36^. Our shrubby model community was adequate for our purpose because it is species-poor, has low plant cover, and the dynamics and structure of the whole community are dominated by the two focal species, *Helianthemum squamatum* (L.) Dum. Cours. (Cistaceae, 41.3% of total plant cover) and *Lepidium subulatum* L. (Brassicaceae, 16.2% of total plant cover). Both are gypsum specialists, and drought is critical for their population dynamics and for the whole plant community dynamics^20,31^. Soil surface is covered by a biological soil crust dominated by lichens interspersed with open areas^37^ and median shrub cover is 30%. Sensitivity to drought may differ among species in these gypsum environments^38^, triggering potential shifts at the community level^39^.

We developed a multispecies dynamic IPM in which vital rates of interacting species were yearly updated depending on climate and on variations in intra- and interspecific covers to assess three specific hypotheses: (1) common climatic constraints have contrasting effects on different vital rates and drive idiosyncratic demographic responses in coexisting species; (2) intra- and interspecific biotic interactions modulate the demography of interacting species, and (3) the intensity and direction (i.e*.* competition and facilitation) of reciprocal interactions vary with climate fluctuations. To test our hypotheses, we used nine years of demographic data and previous knowledge about the dynamics of these semiarid plant communities^33,35,36,40–45^ to parameterize demographic models and to simulate population dynamics of the two focal species under different climatic and ecological scenarios.

## Results

During the nine years of demographic sampling, from 2004 to 2012, we monitored 6290 and 1200 adult plants, and 9182 and 3202 seedlings of *H. squamatum* and *L. subulatum*, respectively. During the whole study period, only 22% of *H. squamatum* seedlings survived their first summer and transited into adults; in *L. subulatum* the rate was even lower (12%). Average seedling emergence showed sharp inter-annual variation, ranging from 0.24 (in the extremely dry year 2008) to 137.5 seedlings m^−2^ (in 2004) in *H. squamatum*, and from 0.08 (in 2008 and 2011) to 61.4 seedlings m^−2^ (in 2006) in *L. subulatum*. Seedling survival ranged from 1% (in 2006 and 2009) to 100% (in 2011) in *H. squamatum*, and from 0% (from 2004 to 2009) to 100% (in 2011) in *L. subulatum.*

### Effects of climate and plant–plant interactions on vital rates

**Figure 1:**
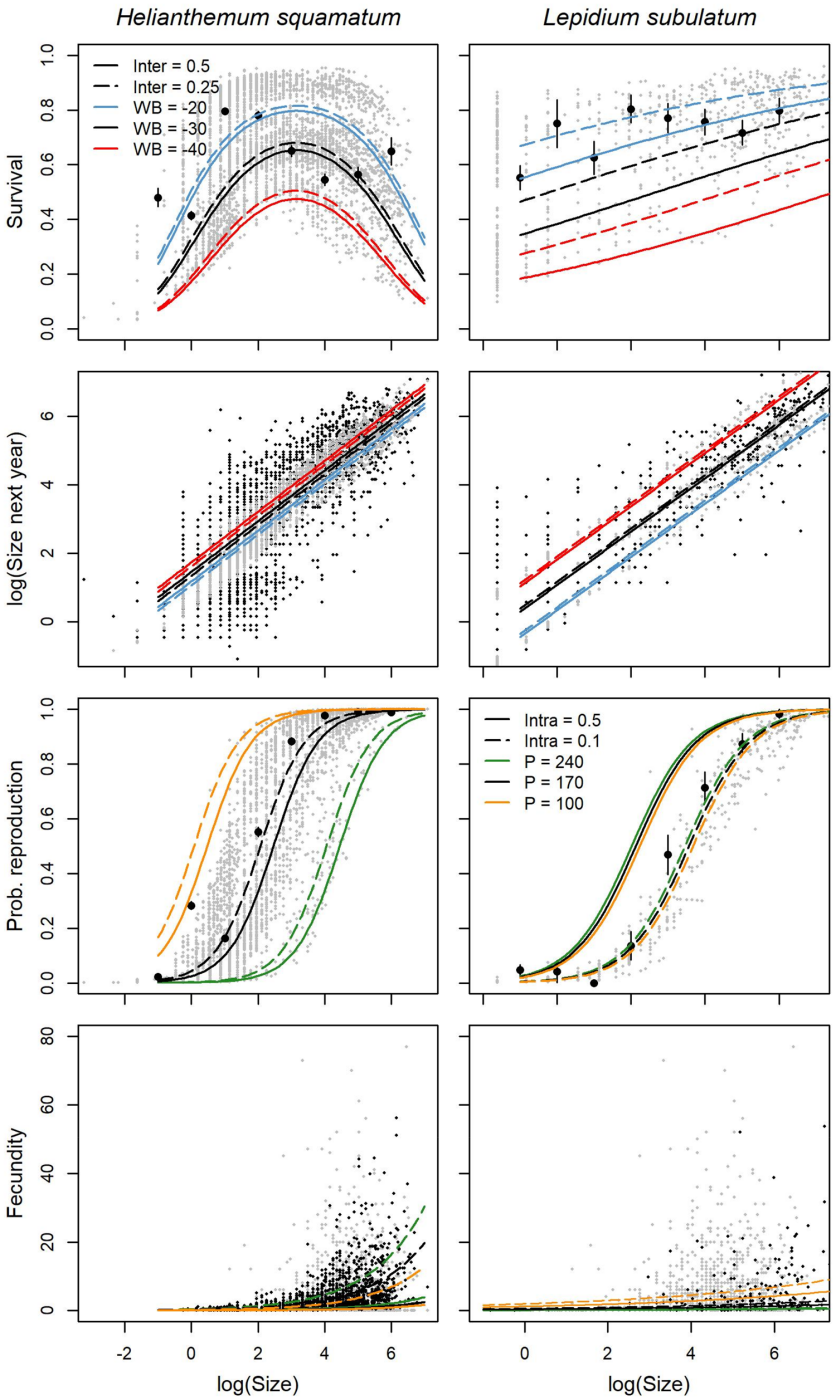
Relationships between plant size and vital rates (survival, growth, probability of reproduction, fecundity) in *H. squamatum* and *L. subulatum* modelled with GLMMs. Black dots are observed values, mean values with standard errors in the cases or survival and probability of reproduction. Grey dots represent predicted values. Lines represent variations in adjusted values related to variations in two of the explanatory variables included in the most explanatory model selected by species and vital rate. *Intra* intraspecific interaction index, *Inter* interspecific interactions index, *WB* summer water balance, *P* spring rainfall.

We followed a three-step procedure to develop our demographic model. As a first step, we explored the effects of climate (summer water balance, spring rainfall and winter minimum temperature) and densities of each species on their vital rates (i.e. survival, growth, probability of reproduction and fecundity) using generalised linear mixed-effect models (GLMMs). Survival and growth showed similar responses to climate in both species, with higher survival but lower growth rates associated to cold winters, rainy springs and wet summers (Table 1, Fig. 1, Supplementary Tables S1–S8). However, warm winters and dry springs increased the probability of reproduction in *H. squamatum*, but not in *L. subulatum*. Fecundity was enhanced by cold winters and wet summers in both species, although spring rainfall had contrasting effects, increasing fecundity in *H. squamatum* but marginally decreasing it in *L. subulatum*. Conspecifics favoured survival in *H. squamatum* but had negative effects on the other vital rates, whereas only probability of reproduction (positively) and fecundity (negatively) were affected by conspecifics in *L. subulatum* (Fig. 1). Effects of interspecific interactions were also evident: *L. subulatum* favoured *H. squamatum* growth, whereas *H. squamatum* decreased survival and fecundity in *L. subulatum*. Since we sampled in two areas with contrasting grazing intensity (blocks A and B), we also included this variability in our models. Probability of reproduction and fecundity in *H. squamatum* were higher in block B, where grazing intensity was lower, but no differences between blocks were found for *L. subulatum* (Table 1).

**Table 1:** Coefficients ± standard error of fixed explanatory variables (excluding size) considered in the most informative GLMMs for vital rates of the study species.

	Biotic interactions		Climate			Block B
	Intra-specific	Inter-specific	Winter temperature^a^	Spring rainfall^b^	Summer water balance^c^	
***H. squamatum***						
Survival	**1.116 ± 0.268**	− 0.481 ± 0.359	− **0.627 ± 0.069**	**0.014 ± 0.001**	**0.073 ± 0.004**	− 0.091 ± 0.091
Growth	− **0.722 ± 0.145**	***0.454 ± 0.170***	***0.075 ± 0.038***	**-0.005 ± 0.001**	− **0.028 ± 0.002**	− 0.021 ± 0.059
Reproduction	− **1.471 ± 0.408**	*0.872* ± *0.500*	**2.510 ± 0.114**	− **0.042 ± 0.002**	–	**0.507 ± 0.147**
Fecundity	− **4.731 ± 0.181**	0.154 ± 0.102	− **1.093 ± 0.022**	**0.016 ± 0.000**	**0.054 ± 0.002**	***0.365 ± 0.162***
***L. subulatum***						
Survival	0.454 ± 0.893	**− 2.030 ± 0.611**	− **0.690 ± 0.200**	*0.005* ± *0.003*	**0.084 ± 0.009**	–
Growth	–	− 0.363 ± 0.312	**0.622 ± 0.076**	− **0.009 ± 0.001**	− **0.074 ± 0.005**	–
Reproduction	***4.511 ± 1.922***	–	–	–	–	0.270 ± 0.412
Fecundity	− ***1.432 ± 0.458***	− ***1.298 ± 0.424***	− **0.532 ± 0.072**	− *0.002* ± *0.001*	**0.158 ± 0.007**	− 0.170 ± 0.304

^a^Averaged minimum temperature from December to February.

^b^Accumulated rainfall from February to May.

^c^Water balance (accumulated precipitation − 2 * temperature) from June to September.

Significant coefficients at *P* < 0.001 and *P* < 0.05 are highlighted in bold and in bold italics, respectively. Marginally significant coefficients (*P* < 0.1) are in italics.

### Effects of climate and plant–plant interactions on population growth rates

**Figure 2:**
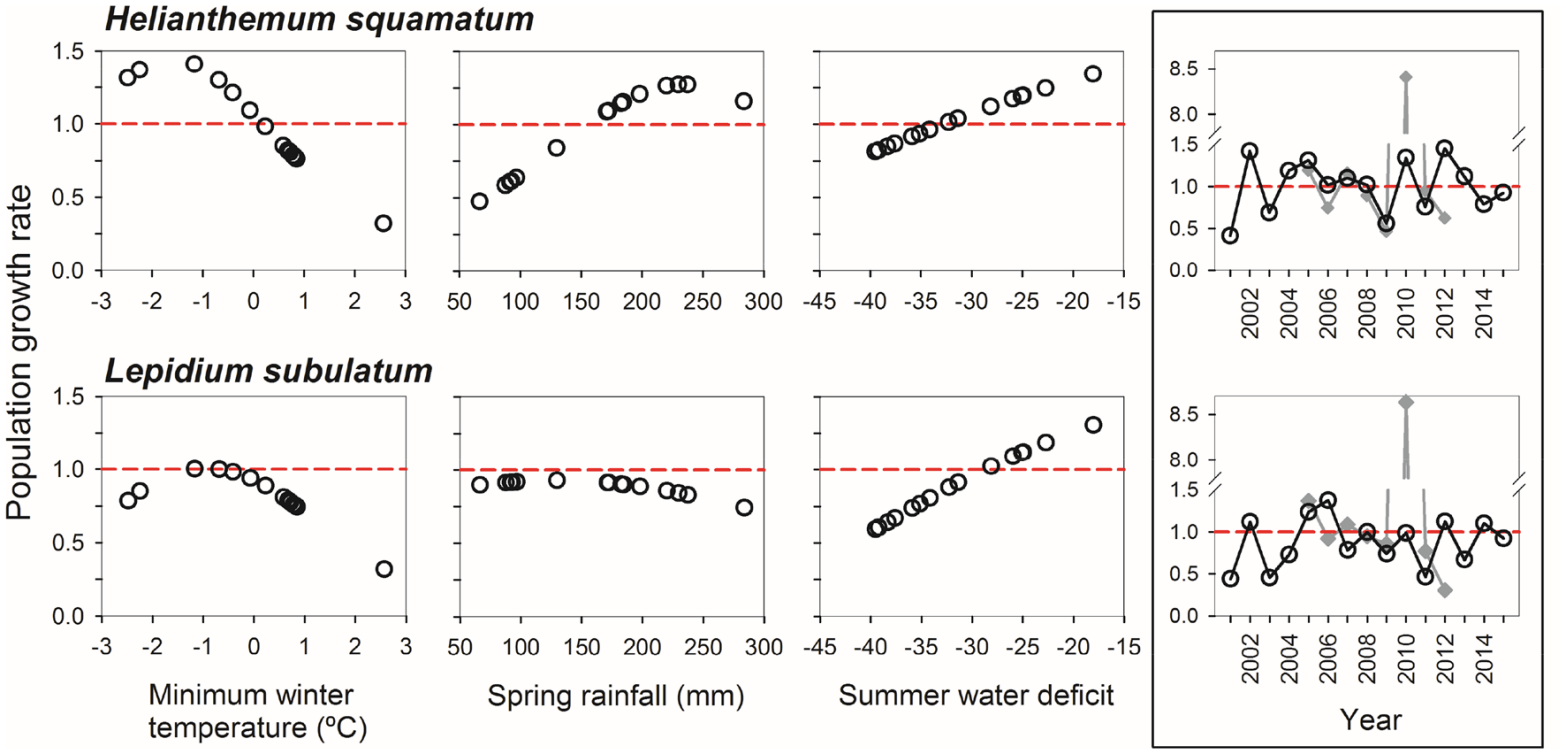
Variation in population growth rate (lambda) with climatic variables (minimum winter temperature, spring rainfall and summer water balance) for *Helianthemum squamatum* and *Lepidium subulatum*. Lambdas were obtained from the Integral Projection Model built for each species separately, adjusting it with the observed values of climatic variables from 2001 to 2015. Models were adjusted for block A and intra- and interspecific covers of 30%; results for block B were similar (see Supplementary Fig. S1). Right hand panels represent population growth rate per year combining the three climatic variables. Note that the highest value of simulated lambda (black line and open symbols) corresponded to 2012 in *H. squamatum* and 2006 in *L. subulatum*, whereas the highest increase in observed population growth rate (grey lines and filled symbols) occurred in 2010 and was much higher than that adjusted by the model.

**Figure 3:**
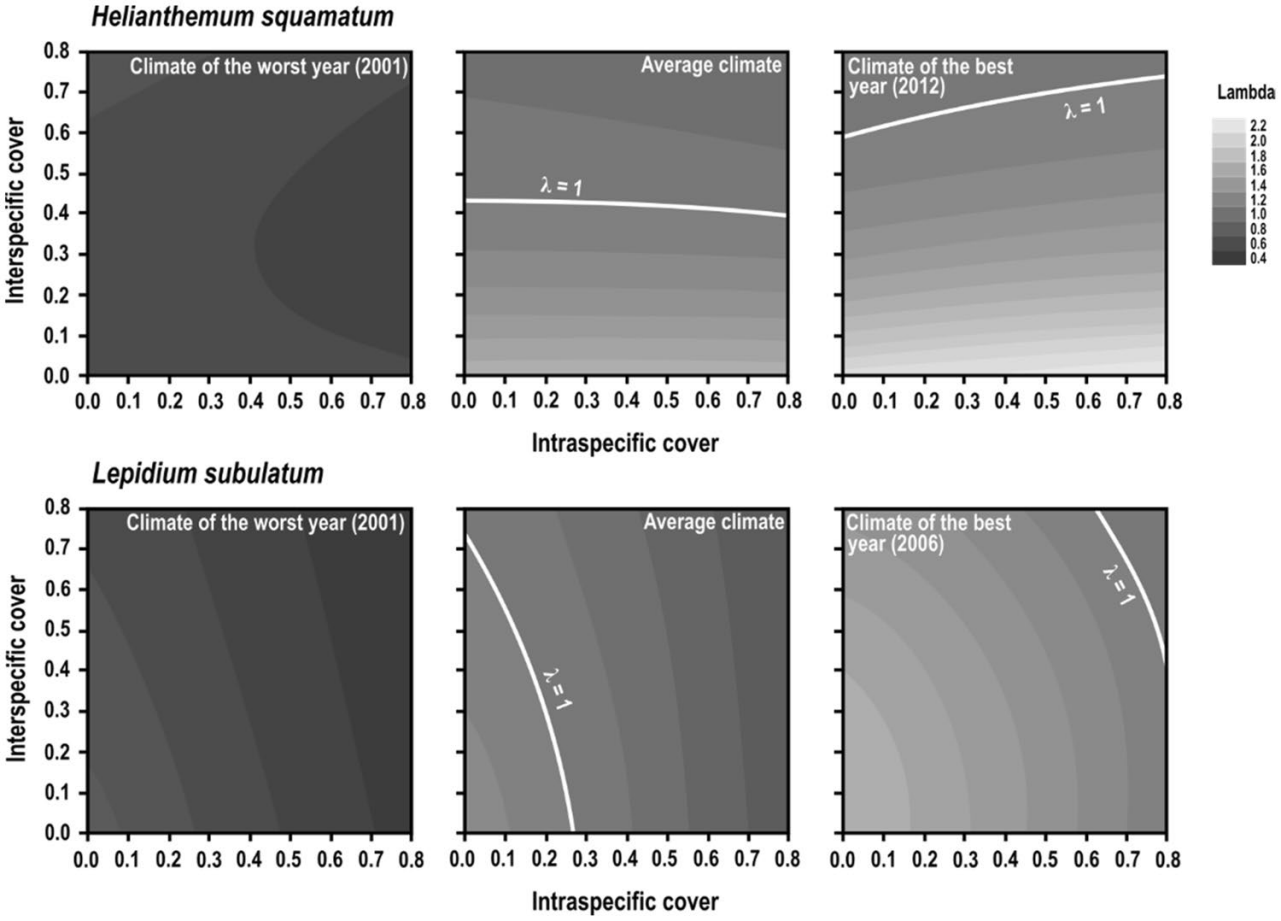
Variation in population growth rate (lambda) depending on intra- and interspecific covers for *H. squamatum* and *L. subulatum*. Lambdas were obtained by running the Integral Projection Models built for each species separately, using contrasting climatic conditions and adjusting intra and interspecific covers in each iteration to cover all possible combinations. White lines indicate the limit between negative (darker) and positive (lighter) population growth rates (i.e., lambda = 1). Models were adjusted for block A; results for block B were similar (see Supplementary Fig. S2). Note that covers of 80% of the two species are unrealistic.

In a second step, we combined the outcome of GLMMs into two integral projection models (IPMs), one per species, to evaluate how variations in climate and in plant densities affected population growth rates (i.e. lambdas). These species-level IPMs showed that population growth rate responded similarly in both species to variations in minimum winter temperature and summer water balance (Fig. 2). Lambda followed a quadratic relationship with minimum winter temperature, *H. squamatum* performing better than *L. subulatum* under colder conditions, and in both species, lambda increased linearly with higher summer water balance. Our predictions of the combined effect of the three climatic variables on lambda were not consistent with estimates of population change based on field counts of standing individuals (Spearman ρ = 0.24 in *H. squamatum*, ρ = 0.19 in *L. subulatum*), mostly due to extremely high increases in plant numbers probably associated with germination from soil seed banks. We used the species-level IPMs to identify which combinations of winter temperature, spring rainfall and summer water balance values had positive or negative effects on population growth rates. Simulated lambdas under the recorded climatic conditions between 2001 and 2015 showed synchrony between both species (Spearman ρ = 0.59), but some differences could be appreciated in certain years, being 2002 and 2012 exceptionally good for *H. squamatum*, whereas 2006 was the best year for *L. subulatum* (Fig. 2). Unfavourable years were more synchronic between species, with strong decreases in lambda in 2001, 2003 and 2011. Regarding biotic interactions, they had significant and asymmetric effects on population dynamics of both species. Intra- and interspecific covers affected population growth rates in a non-linear way, particularly in *L. subulatum* (Fig. 3). Under the average climate conditions of the whole period, low covers of *L. subulatum* increased lambda in *L. subulatum* and *H. squamatum*. In contrast, cover of *H squamatum* only had a slight effect on both lambdas. Years with favourable climatic conditions favoured increases in lambda of both species, whereas unfavourable years reduced lambda. Lambdas were similar between blocks (Supplementary Figs. S1, S2).

### Reciprocal demographic variations under different climatic and ecological scenarios

**Figure 4:**
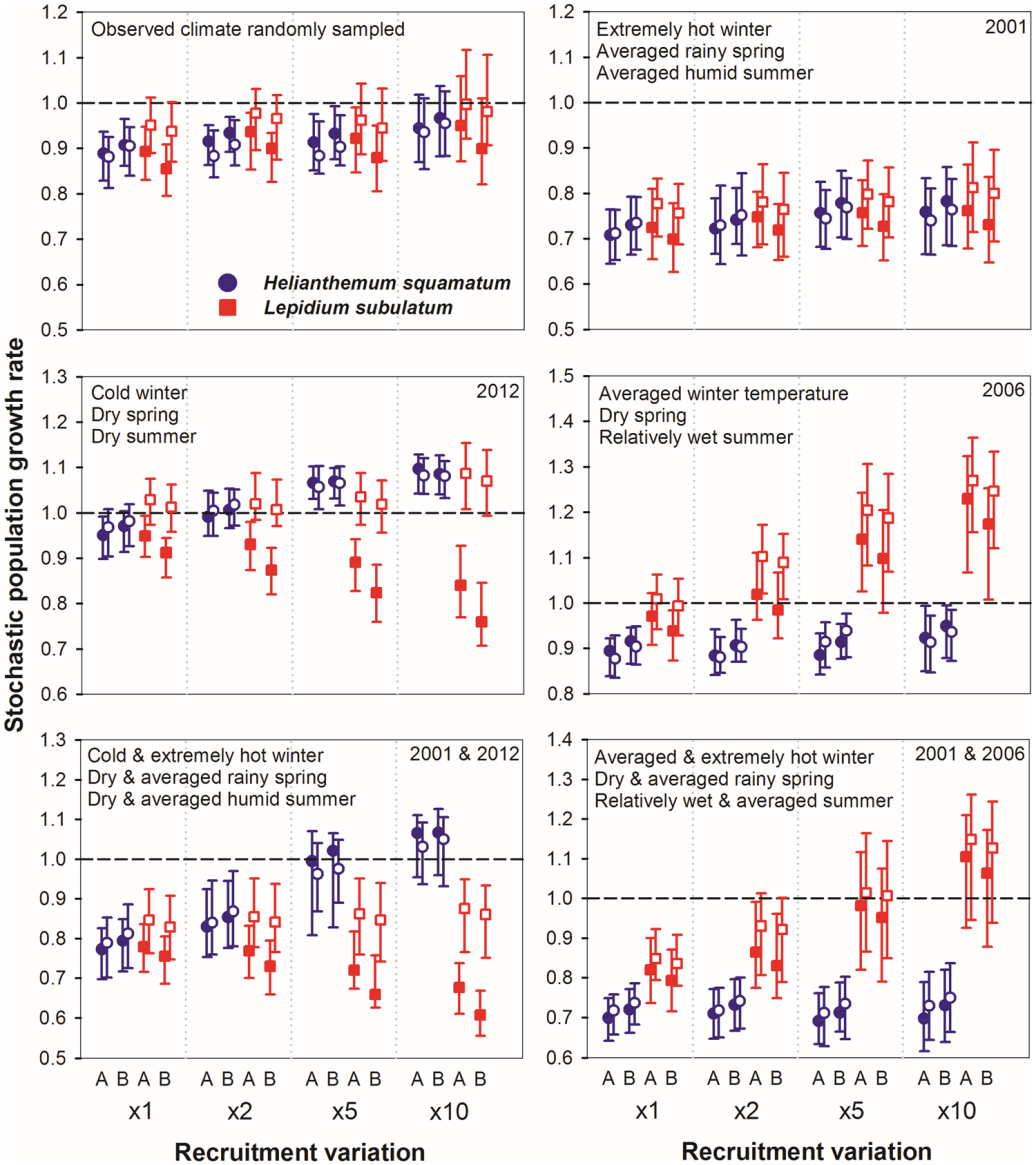
Median and quartiles of stochastic population growth rates obtained from 100 simulations of 14 annual transitions in *Helianthemum squamatum* and *Lepidium subulatum*. Upper panels consider parameters specified in Supplementary Table S9 for simulations 1–4 (left) and 5–8 (right); mid panels consider parameters for simulations 9–12 (left) and 13–16 (right); lower panels correspond to simulations 17–20 (left) and 21–24 (right). Numbers in the upper-right corner indicate the year from which climatic conditions (specified in each panel) were more frequently used. Open symbols represent simulations where the interaction between *H. squamatum* and *L. subulatum* was null, whereas filled symbols represent simulations where the interaction was considered. Stable population growth rate (λ = 1) is marked with a dashed horizontal line in all plots. The letters A and B in the X axis indicate blocks. See Supplementary Table S10 for exact values of climatic variables.

The third step combined the two species-level IPMs to develop a multispecies dynamic IPM in which variations in vital rates of both species readjusted reciprocally depending on changes in plant density of the other species and on environmental conditions. This multispecies dynamic IPM was used to simulate variations in lambdas under scenarios of increased climatic variability (Supplementary Tables S9, S10). Increases in the frequency of favourable years tended to raise stochastic population growth rates in both species (Fig. 4). We linked the occurrence of favourable years with recruitment rises to mimic the “rescue” events typical of the community under study. A twofold rise in recruitment was sufficient to achieve positive lambdas when only the frequency of favourable years increased, whereas the combination of higher frequencies of both favourable and unfavourable years needed a minimum of five or tenfold recruitment rise linked to the occurrence of benign conditions. Increased frequency of unfavourable years lowered lambdas in both species, regardless recruitment intensity. Including dynamic biotic interactions reinforced the non-linear interplay between intra- and interspecific covers. Under this dynamic perspective, *H. squamatum* decreased *L. subulatum* lambda, but the reciprocal relationship did not have clear effects. The negative effect of *H. squamatum* on *L. subulatum* was enhanced as the frequency of favourable years for the former increased, particularly under higher recruitment intensities (Fig. 4).

**Figure 5:**
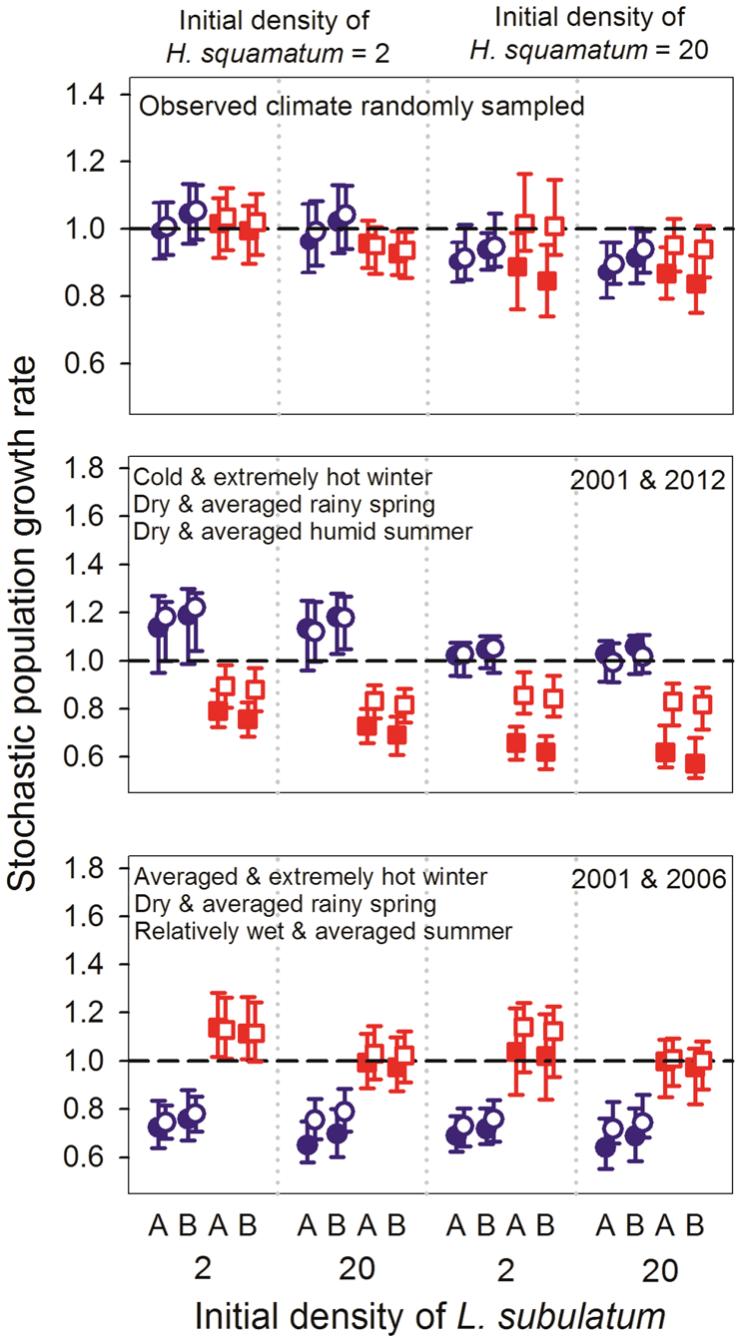
Effect of varying initial densities (2 and 20 individuals m^−2^) of both species on the stochastic population growth rate under different climatic conditions and when recruitment is increased tenfold. Open symbols represent simulations where the interaction between *H. squamatum* and *L. subulatum* was null, whereas filled symbols represent simulations where the interaction was considered. Stable population growth rate (λ = 1) is marked with a dashed horizontal line in all plots. The letters A and B in the X axis indicate blocks. See Supplementary Table S10 for exact values of climatic variables.

To assess whether observed and simulated reciprocal demographic effects under different climatic scenarios were determined by the initial densities of both species, we run additional simulations modifying them (Supplementary Table S9). Increases in initial densities of *L. subulatum* and *H. squamatum* slightly decreased their own lambdas, suggesting the presence of negative density-dependence in both cases (Fig. 5). Finally, the negative effects of *H. squamatum* on *L. subulatum* did not differ between blocks except when the frequency of favourable years for *H. squamatum* increased, in which case the effect was stronger at block B, which had lower trampling intensity.

## Discussion

The development of demographic models of interacting species and the analysis of their demographic trends under simulated climate scenarios evidenced the complexity of population responses of coexisting species to climatic fluctuations. We found that scenarios in which populations of coexisting species responded in an idiosyncratic way to climatic fluctuations^6^, were in fact combined with scenarios in which responses to climate were quite similar. The existence of one or another outcome depended on each year combination of climatic factors, partially validating our first hypothesis. Biotic interactions modulated some of the vital rates of our interacting species, both at intra- and interspecific levels, but their direct effects on population dynamics were less evident than those of climate, partially supporting our second hypothesis. Finally, we observed that the interspecific demographic effects were not symmetrical (i.e., *H. squamatum* emerged as a stronger competitor than *L. subulatum*) and highly depended on climate conditions. Favourable years for *H. squamatum* enhanced its negative effect on *L. subulatum* population dynamics, which validated our third hypothesis. Altogether, these results support the idea that population dynamics are strongly dependent on climatic variations^3,4^, and that a more accurate understanding of climate-related demographic fluctuations undoubtedly needs the explicit consideration of the interplay between the population dynamics of coexisting species.

Agreement in population growth rates of *H. squamatum* and *L. subulatum* in certain years may be explained by the fact that survival and growth responded similarly to climate in both cases. Both species shared positive responses in years with dry springs (e.g., 2005 or 2012, see Supplementary Table S10), probably due to the fact that the diverse community of annual species that emerges in moist years is not able to develop under spring drier conditions^46–48^, thus reducing competition with our shrubby target species. Otherwise, contrasting population responses observed in certain years might be explained by their differences in the response of fecundity to spring rainfall: the late-flowering *H. squamatum* increased its fecundity in rainy springs, whereas *L. subulatum* showed the opposite pattern. This probably reflects the earlier blooming phenology of *L. subulatum* (Supplementary Table S11), as abundant rains during the flowering period may lead to pollination failure^49^, negatively affecting population dynamics.

Species-level demographic models (see Fig. 2) were not able to reproduce observed variation in population growth rates, particularly in 2010, when a strong recruitment peak was recorded. This was an extremely wet year, with 524 mm of total annual rainfall and 237 mm of them (i.e. 45%) falling between February and May. Massive recruitments due to pulse dynamics of resource availability are well known in semiarid and arid conditions, where water availability is one of the most important determinants^50,51^. In fact, although small and continuous recruitment events are also needed for population persistence^50,52^, extreme recruitment pulses are critical for guaranteeing persistence in the long term. A plausible explanation for the poor reproduction of this pulse event—and for the poor fit between observed change in standing plants and simulated lambda—is that our sampling methods did not distinguish between germinant seeds coming from the seed bank and seeds coming from direct adult production. Both study species have a persistent and dynamic seed bank that is particularly dense in *H. squamatum*^43,53,54^. An adequate inclusion of pulse dynamics in demographic models would therefore require modelling those factors that determine massive germination and recruitment from the soil seed bank. We partially circumvented this limitation in the multispecies dynamic model by including recruitment rises in our simulations, and in fact, we obtained positive stochastic population growth rates when recruitment in favourable years rose five or tenfold. Otherwise, the fact that 2010 was the only year in which the recruitment pulse was recorded, despite other years, such as 2004, 2007 or 2013, also having similar spring rainfall amounts, suggests that not only spring rainfall, but its combination with other climatic drivers such as summer water balance (which, actually, was the least negative in 2010) may be responsible for recruitment pulses. The modulating effects of population dynamics of interacting species may also intervene in pulse dynamics, but this needs to be further explored.

Our multispecies dynamic model revealed an unexpected asymmetric effect of interspecific interactions. The inclusion of interspecific interactions (i.e*.* of reciprocal demographic effects between the two study species) in the multispecies dynamic model showed that the stochastic population growth rate of *H. squamatum* was less affected by the dynamics of *L. subulatum* population, despite clear effects of *L. subulatum* had been previously observed in individual growth rate (positive) and in static population growth rate of *H. squamatum* (negative, see Fig. 3). This paucity of net interspecific effects of *L. subulatum* on *H. squamatum* dynamics may be the result of different positive and negative effects cancelling each other out^17,18,22^, leading to a net neutral interaction. But it may also reflect the ability of *H. subulatum* to adjust reproduction and resource allocation in stressful conditions to maximise its fitness^52^. In contrast, *H. squamatum* had a strong negative effect on *L. subulatum* demography. This negative effect was enhanced in years when climate favoured *H. squamatum* population growth, therefore suggesting that *H. squamatum* was a stronger competitor. Asymmetries in interspecific relationships between plants have been reported among coexisting species in terms of impacts on their performance or fitness (e.g.^55,56^), and our multispecies dynamic model adds on the impact of those asymmetries on population dynamics.

According to the contemporary coexistence theory, relative fitness differences (i.e. differential performance of populations under specific conditions) drive competitive dominance among species, whereas niche differences prevent competitive exclusion. Within this framework, stable coexistence is promoted when stabilising mechanisms (i.e. niche differences) predominate over fitness differences^5,57^. Our approach, based on demographic empirical information and multispecies dynamic models, provided insights on the interplay between stabilising niche and relative fitness differences. This demographic approach relies on the premise that stabilising niche differences influence coexistence by causing species to have greater intrinsic population growth rates when they are rare^57,58^. In other words, the presence of negative density-dependence in population dynamics is a signature of stabilising niche differences^5^. Our simulations suggested the existence of negative density-dependence in both species, which may be assumed as an indicator of stabilising niche differences between them. On the other hand, the competitive dominance of *H. squamatum* points to the existence of relative fitness differences. Although our approach did not allow to quantify the relative importance of both mechanisms for species coexistence, competitive dominance of *H. squamatum* was evident when the frequency of years with favourable climatic conditions increased. This fact hints the existence of some buffering mechanism in *L. subulatum* that allows it to maintain the population in years when it is experiencing stronger competition levels. These mechanisms may be related to the higher life expectancy of *L. subulatum* (more than 20 years^20,36^) compared with *H. squamatum* (between 4 and 6 years^33^), or to its summer deciduousness, which reduces the activity of adult plants in summer and makes them relatively immune to unfavourable environmental and competitive conditions. In any case, our results clearly show that increased levels of climatic variability and higher frequency of occurrence of extreme climatic events, as predicted by many climate models, might alter interspecific relationships and disrupt competitive hierarchies, leading to abrupt changes in community composition.

Through this demographic approach we demonstrate that plant population dynamics are regulated by abiotic conditions and modulated by plant to plant interactions. Other factors such as soil characteristics, livestock grazing pressure or biodiversity variations at fine scale may also modulate the responses of plant species to climate^8–10^ and should certainly be considered in further demographic studies. In any case, our multispecies model attempted to link plant population ecology with community ecology. From this perspective, natural assemblages are just a snapshot of interacting populations and we need a demographic perspective and user-friendly tools to advance in the construction of a definitive coexistence theory^5,59^. We recognize that a plant community is usually composed of more than two interacting species, but in our case, as with other relatively simple communities occurring in harsh environments, a small number of species structure the whole community and drive its dynamics^40,41^. The explicit consideration of plant to plant interactions and their environmental context on demographic models would therefore lead to a better understanding of the cascading ecological and evolutionary effects that climate change might have at the community level^60^.

## Methods

### Study area and fieldwork

Data were collected in Belinchón (40° 3′ 20′′ N, 3° 3′ 31′′ W, 720 m a.s.l.), located in the evaporite-bearing unit of Tajo-Mancha basin, in central Spain^61^. This is a semi-arid region with annual rainfall amounts of approximately 441 mm on average and extreme summer droughts (only 5.6% of the annual rainfall occurs during July and August). Soils are gypsisols, developed over massive gypsum parental rocks^62^. We selected two areas of 20 × 20 m (hereafter blocks A and B) located 300 m apart. Both areas are grazed by sheep but grazing intensity is slightly higher in block A (A. Escudero, pers. obs.). Each block was divided into a 2 × 2 m grid, resulting in 1004 m^2^ cells per block. We selected half of the cells following a checkerboard pattern and established a 0.5 × 0.5 m sampling plot in the centre of each one, thus obtaining 50 plots per block. All *H. squamatum* and *L. subulatum* plants within the plots were marked and monitored twice a year, in April and September (given that summer is the most critical period for seedling survival and adults’ growth^40,41^), between April 2004 and April 2012. At each sampling date, living status (alive or dead) and size (height and two perpendicular crown diameters) were recorded for each individual. Flowering status was recorded in April for *L. subulatum*, using a qualitative estimate of relative flowering intensity with four levels (none, low, medium and high), and in September for *H. squamatum* based on any evidence of present inflorescences (i.e., flowering or not). Emerged seedlings were counted during April sampling, when emergence reaches its peak. Differences between life cycles of both target species are summarised in Supplementary Methods M1 and Table S11.

### Calculation of vital rates

We obtained annual survival, growth, probability of reproduction and fecundity in both species. Growth was calculated as the size difference between a given year and the next year, defining plant size as crown projected area in cm^2^ estimated from the mean crown diameter. Probability of reproduction was defined as a binary variable considering whether an individual flowered or not. Fecundity per plant was estimated from the number of observed seedlings per plot, assigning them to adults in proportion to plant size (in *H. squamatum*) and in proportion to plant size and flowering degree (60%, 30% and 10% for high, medium and low flowering degree, respectively) in *L. subulatum*. We assumed that seed production depended on plant cover per year; this method has some drawbacks, since we cannot distinguish between germinant seeds coming from the seed bank and seeds coming from direct adult production. However, our fecundity estimates for *H. squamatum* were commensurate (but slightly lower) than those observed in a nearby gypsum locality where seed production per plant and seed bank dynamics have been recorded^45^. It is worthy to note that both species have seeds with mucilaginous coats that anchor them onto the soil surface in the immediate vicinity of mother plants^40,41^, leading to a strong small-scale pattern of seed availability^43^.

### Climatic data and biotic interactions

Climatic data for the period 2004–2011 were obtained from the Barajas de Melo meteorological station, located 15 km away from the study area (Spanish Ministry of Agriculture, Food and Environment, http://crea.uclm.es/siar/datmeteo/, Supplementary Table S10). Given the importance of summer drought for our study species^33,36^, we selected summer water balance (i.e., *P –* 2*T*, where *P* is accumulated rainfall and *T* is mean temperature from June to September) and accumulated spring rainfall (from February to May) as the most informative climatic variables. Winter minimum temperature (from December to February) was also included, as low temperatures arrest plant growth and development.

Biotic interactions were estimated as indices per plot. Maximum root spread in both species (60.7 cm in *H. squamatum*, 56.9 cm in *L. subulatum*^63^) indicates that the maximum distance at which individuals may interact is around 60 cm. As all individuals within a plot were located at a maximum distance of 70 cm from each other, this scale seems adequate to estimate interaction effects. Intraspecific interaction for a given plant was assessed as the sum of covers of all individuals of the same species within the plot, excluding the focal plant. Interspecific interaction per plant was similarly obtained as the sum of covers of all individuals of the other target species within the plot (i.e. only considering the cover of *H. squamatum* or *L. subulatum*, and excluding all other species in the community).

### Modelling of vital rates (model step 1)

We fitted generalised linear mixed-effects models (GLMMs) to assess the response of annual survival, growth, probability of reproduction and fecundity to climate (summer water balance, spring rainfall, winter minimum temperature) and biotic interactions (intra- and interspecific indices) in both species. In all models, plant size, block, climate and biotic interactions were included as fixed explanatory variables, and plot was considered as a random factor. We compared a battery of models including plant size and additive combinations of the rest of explanatory variables, and selected the most plausible model using the Akaike Information Criterion (AIC). When different models had ΔAIC lower than 2, they were considered similar and the most parsimonious model was chosen. When block and the interspecific interaction appeared within the most plausible models, both of them were included in the selected model, since block was part of the design and the interspecific interaction was a main focus in the study. Survival models were parameterized using a logit link and binomial error distribution, considering a quadratic relationship with plant size. Growth was modelled as a function of size in the previous year, with identity link and Gaussian error distribution. Probability of flowering was modelled with the logit link and binomial error distribution, excluding summer water balance from the explanatory variables given that both species start flowering before June. Fecundity models were parameterized with logarithm link and Poisson error distribution (see Supplementary Tables S1–S8 for GLMMs specification and selection). Selected models for each vital rate were readjusted with restricted maximum likelihood. To fit GLMMs, we used the R packages *lme4*^64^ and *nlme*^65^.

### Species-level IPMs (model step 2) to characterise population growth rates

We modified code from the *IPMPack* package (version 1.5^66^) in R (version 3.2.2^67^) to build an integral projection model (IPM) per species. We used the selected GLMMs for survival, growth, probability of reproduction and fecundity to derive our kernel function^27^:

$$n(y,t+1)={\int }_{T}^{U}\left[s\left(x,y\right)g\left(x,y\right)+f\left(x,y\right)\right] n\left(x,t\right)dx,$$

where the probability function of individuals at time *t* + 1 (*n*(*y,t* + 1)) is equal to the integration of survival (*s(x,y)*), growth (*g(x,y)*) and reproduction (*f(x,y)*) across the possible range of sizes (*T* to *U*). We implemented the IPMs by applying the mid-point rule to discretise the integration^28^, with a final matrix size for each IPM of 200 × 200 cells.

We used these IPMs to characterise the relationship between population growth rates of both species and climatic variables for the period when climatic data were available (2000 to 2015). The range of values of relative cover per species (from 0 to 1) was also used to characterise the response of population growth rates to intra- and interspecific interactions under contrasting climatic conditions. Specifically, we ran the IPMs using average climate data for the whole period (2000–2015) and also using the climatic conditions of years when population growth rates were the lowest (2001) and the highest (2006 for *L. subulatum*, 2012 for *H. squamatum*).

### Multispecies dynamic IPM (model step 3) and demographic simulations

Species-level IPMs were combined into a single model in which yearly values of intra- and interspecific covers updated every iteration. To this end, the abundance of each of the 200 size classes in the population vector was multiplied by the midpoint of the cover of each class per iteration, and yearly matrices were then multiplied by population vectors to estimate the abundance matrix in the following year. Climatic data per iteration were combined with information of both species’ cover to predict their vital rates. This multispecies dynamic IPM was used to simulate stochastic population growth rates of both species under different climatic and ecological scenarios. We simulated 14 transitions (15 years) in order to adjust to the length of the period with observed climatic data (2000–2015). The starting point for each simulation was the vector of abundances by cover class observed during the first year of study (2004). We run 100 replicated simulations per scenario, recording each time plant abundance by year and using it to calculate the stochastic population growth rate for the whole period per species and block.

To define climatic scenarios, we used the 2000–2015 climatic dataset. Climatic conditions per iteration were randomly selected from this dataset according to the following rules: (1) random climatic variation within the observed ranges; (2) an increase in the frequency of climatically favourable years detected with species-level IPMs in such a way that 1 of every 4 years corresponded with climate in 2006 for *L. subulatum* and 1 of every 3 years corresponded with climate in 2012 for *H. squamatum*; (3) an increase in the frequency of climatic conditions related to unfavourable years (i.e., 1/3 of years corresponded with climate in 2001), and (4) a combination of increases in the frequency of both favourable and unfavourable conditions. Ecological scenarios included (1) variations in initial densities of both species according to the observed range in 2004 (2.4 and 16 individuals m^−2^ for *L. subulatum* and *H. squamatum*, respectively), considering 2 and 20 individuals m^−2^ and their possible combinations (i.e., 2–2, 2–20, 20–2 and 20–20 individuals m^−2^) as initial densities for both species, and (2) variations in recruitment to mimic pulse events, which were defined as two, five and tenfold increases in recruitment during favourable years^43,54^.

Finally, to evaluate the demographic impacts of one species on the other, all simulations were done twice: (1) iteratively recalculating covers of both species based on their demographic responses, and (2) cancelling the interspecific effect when recalculating covers for the following iteration (see Supplementary Table S9 for details on simulations’ definition). All simulations were also independently replicated per block in order to assess potential differences due to the local context.

## Supplementary information

Supplementary information.

## Data Availability

The demographic datasets analysed during the current study are available in the Biodiversos-URJC repository, http://repositories.biodiversos.org/Garcia-Cervigon.Ana_I.
