# Supplementary material for: Demographic effects of interacting species: exploring stable coexistence under increased climatic variability in a semiarid shrub community
Source: Sci Rep. 2021 Feb 4;11:3099. doi: 10.1038/s41598-021-82571-z (PMC7862631; doi:10.1038/s41598-021-82571-z)
Supplement: Supplementary file 1 — Supplementary information. [file 41598_2021_82571_MOESM1_ESM.docx]

***Supplementary Information***

**Demographic effects of interacting species: exploring stable coexistence under increased climatic variability in a semiarid shrub community**

Ana I. García-Cervigón, Pedro F. Quintana-Ascencio, Adrián Escudero, Merari E. Ferrer-Cervantes, Ana M. Sánchez, José M. Iriondo and José Miguel Olano

**Supplementary results**

**Table S1.** AIC table of GLMMs for *H. squamatum* survival. All models were adjusted with the function *glmer()* in package *lme4* (Bates et al. 2014), including plot as random factor and using binomial error distribution. K: number of estimated parameters for each model; AICc: Akaike weights. Abbreviations for explanatory variables: WDJS: water deficit from June to September; PFM: rainfall from February to May; TmDF: average minimum temperature from December to February; Intra: intraspecific interaction index; Inter: interspecific interaction index. Selected model is highlighted in bold.

| Model | K | AICc | ΔAICc |
| --- | --- | --- | --- |
| Surv ~ log(size) + log(size)^2^ + Intra + WDJS + PFM + TmDF | 8 | 5995.022 | 0.000 |
| Surv ~ log(size) + log(size)^2^ + Intra + Inter + WDJS + PFM + TmDF | 9 | 5995.421 | 0.399 |
| Surv ~ log(size) + log(size)^2^ + Intra + block + WDJS + PFM + TmDF | 9 | 5996.199 | 1.177 |
| **Surv ~ log(size) + log(size)^2^ + Intra + Inter + block + WDJS + PFM + TmDF** | **10** | **5996.438** | **1.416** |
| Surv ~ log(size) + log(size)^2^ + Inter + WDJS + PFM + TmDF | 8 | 6009.286 | 14.264 |
| Surv ~ log(size) + log(size)^2^ + WDJS + PFM + TmDF | 7 | 6009.734 | 14.712 |
| Surv ~ log(size) + log(size)^2^ + Inter + block + WDJS + PFM + TmDF | 9 | 6011.053 | 16.031 |
| Surv ~ log(size) + log(size)^2^ + block + WDJS + PFM + TmDF | 8 | 6011.603 | 16.581 |
| Surv ~ log(size) + log(size)^2^ + Intra + WDJS + PFM | 7 | 6078.294 | 83.272 |
| Surv ~ log(size) + log(size)^2^ + Intra + Inter + WDJS + PFM | 8 | 6078.964 | 83.942 |
| Surv ~ log(size) + log(size)^2^ + Intra + block + WDJS + PFM | 8 | 6079.110 | 84.088 |
| Surv ~ log(size) + log(size)^2^ + Intra + Inter + block + WDJS + PFM | 9 | 6079.940 | 84.918 |
| Surv ~ log(size) + log(size)^2^ + WDJS + PFM | 6 | 6087.018 | 91.996 |
| Surv ~ log(size) + log(size)^2^ + Inter + WDJS + PFM | 7 | 6088.287 | 93.266 |
| Surv ~ log(size) + log(size)^2^ + block + WDJS + PFM | 7 | 6088.636 | 93.614 |
| Surv ~ log(size) + log(size)^2^ + Inter + block + WDJS + PFM | 8 | 6089.984 | 94.962 |
| Surv ~ log(size) + log(size)^2^ + Intra + Inter + WDJS + TmDF | 8 | 6158.297 | 163.275 |
| Surv ~ log(size) + log(size)^2^ + Intra + Inter + block + WDJS + TmDF | 9 | 6159.598 | 164.576 |
| Surv ~ log(size) + log(size)^2^ + Intra + WDJS + TmDF | 7 | 6159.934 | 164.912 |
| Surv ~ log(size) + log(size)^2^ + Intra + block + WDJS + TmDF | 8 | 6161.021 | 165.999 |
| Surv ~ log(size) + log(size)^2^ + Intra + Inter + WDJS | 7 | 6163.587 | 168.566 |
| Surv ~ log(size) + log(size)^2^ + Intra + WDJS | 6 | 6164.342 | 169.320 |
| Surv ~ log(size) + log(size)^2^ + Intra + Inter + block + WDJS | 8 | 6164.988 | 169.966 |
| Surv ~ log(size) + log(size)^2^ + Intra + block + WDJS | 7 | 6165.564 | 170.542 |
| Surv ~ log(size) + log(size)^2^ + Inter + WDJS + TmDF | 7 | 6169.806 | 174.785 |
| Surv ~ log(size) + log(size)^2^ + WDJS + TmDF | 6 | 6170.468 | 175.446 |
| Surv ~ log(size) + log(size)^2^ + Inter + block + WDJS + TmDF | 8 | 6171.682 | 176.660 |
| Surv ~ log(size) + log(size)^2^ + block + WDJS + TmDF | 7 | 6172.245 | 177.223 |
| Surv ~ log(size) + log(size)^2^ + WDJS | 5 | 6176.984 | 181.962 |
| Surv ~ log(size) + log(size)^2^ + Inter + WDJS | 6 | 6177.249 | 182.227 |
| Surv ~ log(size) + log(size)^2^ + block + WDJS | 6 | 6178.855 | 183.833 |
| Surv ~ log(size) + log(size)^2^ + Inter + block + WDJS | 7 | 6179.183 | 184.161 |
| Surv ~ log(size) + log(size)^2^ + Intra + PFM + TmDF | 7 | 6331.627 | 336.606 |
| Surv ~ log(size) + log(size)^2^ + Intra + Inter + PFM + TmDF | 8 | 6331.940 | 336.918 |
| Surv ~ log(size) + log(size)^2^ + Intra + block + PFM + TmDF | 8 | 6332.845 | 337.823 |
| Surv ~ log(size) + log(size)^2^ + Intra + Inter + block + PFM + TmDF | 9 | 6332.956 | 337.934 |
| Surv ~ log(size) + log(size)^2^ + Inter + PFM + TmDF | 7 | 6345.372 | 350.350 |
| Surv ~ log(size) + log(size)^2^ + PFM + TmDF | 6 | 6346.025 | 351.003 |
| Surv ~ log(size) + log(size)^2^ + Inter + block + PFM + TmDF | 8 | 6347.172 | 352.150 |
| Surv ~ log(size) + log(size)^2^ + block + PFM + TmDF | 7 | 6347.940 | 352.918 |
| Surv ~ log(size) + log(size)^2^ + Intra + Inter + PFM | 7 | 6538.703 | 543.681 |
| Surv ~ log(size) + log(size)^2^ + Intra + Inter + block + PFM | 8 | 6539.659 | 544.637 |
| Surv ~ log(size) + log(size)^2^ + Intra + PFM | 6 | 6540.998 | 545.976 |
| Surv ~ log(size) + log(size)^2^ + Intra + block + PFM | 7 | 6541.586 | 546.564 |
| Surv ~ log(size) + log(size)^2^ + Inter + PFM | 6 | 6548.074 | 553.052 |
| Surv ~ log(size) + log(size)^2^ + PFM | 5 | 6549.284 | 554.262 |
| Surv ~ log(size) + log(size)^2^ + Inter + block + PFM | 7 | 6549.842 | 554.821 |
| Surv ~ log(size) + log(size)^2^ + block + PFM | 6 | 6550.859 | 555.837 |
| Surv ~ log(size) + log(size)^2^ + Intra + Inter + TmDF | 7 | 6764.370 | 769.348 |
| Surv ~ log(size) + log(size)^2^ + Intra + Inter + block + TmDF | 8 | 6765.852 | 770.831 |
| Surv ~ log(size) + log(size)^2^ + Intra + TmDF | 6 | 6772.215 | 777.193 |
| Surv ~ log(size) + log(size)^2^ + Intra + block + TmDF | 7 | 6773.301 | 778.279 |
| Surv ~ log(size) + log(size)^2^ + Inter + TmDF | 6 | 6777.874 | 782.852 |
| Surv ~ log(size) + log(size)^2^ + Inter + block + TmDF | 7 | 6779.866 | 784.844 |
| Surv ~ log(size) + log(size)^2^ + TmDF | 5 | 6783.967 | 788.945 |
| Surv ~ log(size) + log(size)^2^ + block + TmDF | 6 | 6785.837 | 790.815 |
| Surv ~ log(size) + log(size)^2^ + Intra + Inter | 6 | 6789.915 | 794.893 |
| Surv ~ log(size) + log(size)^2^ + Intra + Inter + block | 7 | 6791.571 | 796.549 |
| Surv ~ log(size) + log(size)^2^ + Intra | 5 | 6795.220 | 800.198 |
| Surv ~ log(size) + log(size)^2^ + Intra+block | 6 | 6796.585 | 801.564 |
| Surv ~ log(size) + log(size)^2^ + Inter | 5 | 6807.087 | 812.065 |
| Surv ~ log(size) + log(size)^2^ + Inter + block | 6 | 6809.088 | 814.066 |
| Surv ~ log(size) + log(size)^2^ | 4 | 6810.511 | 815.489 |
| Surv ~ log(size) + log(size)^2^ + block | 5 | 6812.491 | 817.469 |
| Surv ~ 1 | 2 | 7019.628 | 1024.606 |
| Surv ~ log(size) | 3 | 7020.450 | 1025.428 |

**Table S2.** AIC table of GLMMs for *H. squamatum* growth. All models were adjusted with the function *lme()* in package *nlme* (Pinheiro et al. 2015), including plot as random factor and using Gaussian error distribution. K: number of estimated parameters for each model; AICc: Akaike weights. Abbreviations for explanatory variables: WDJS: water deficit from June to September; PFM: rainfall from February to May; TmDF: average minimum temperature from December to February; Intra: intraspecific interaction index; Inter: interspecific interaction index. Selected model is highlighted in bold.

| Model | K | AICc | ΔAICc |
| --- | --- | --- | --- |
| log(sizeNext) ~ log(size) + Intra + Inter + WDJS + PFM + TmDF | 9 | 9697.475 | 0.000 |
| **log(sizeNext) ~ log(size) + Intra + Inter + block + WDJS + PFM + TmDF** | **10** | **9699.351** | **1.876** |
| log(sizeNext) ~ log(size) + Intra + Inter + WDJS + PFM | 8 | 9699.377 | 1.902 |
| log(sizeNext) ~ log(size) + Intra + Inter + block + WDJS + PFM | 9 | 9701.219 | 3.744 |
| log(sizeNext) ~ log(size) + Intra + WDJS + PFM | 7 | 9702.446 | 4.971 |
| log(sizeNext) ~ log(size) + Intra + WDJS + PFM + TmDF | 8 | 9702.771 | 5.296 |
| log(sizeNext) ~ log(size) + Intra + block + WDJS + PFM | 8 | 9704.149 | 6.675 |
| log(sizeNext) ~ log(size) + Intra + block + WDJS + PFM + TmDF | 9 | 9704.480 | 7.006 |
| log(sizeNext) ~ log(size) + Inter + WDJS + PFM | 7 | 9719.706 | 22.231 |
| log(sizeNext) ~ log(size) + Inter + WDJS + PFM + TmDF | 8 | 9720.214 | 22.740 |
| log(sizeNext) ~ log(size) + Inter + block + WDJS + PFM | 8 | 9720.631 | 23.156 |
| log(sizeNext) ~ log(size) + Inter + block + WDJS + PFM + TmDF | 9 | 9721.146 | 23.672 |
| log(sizeNext) ~ log(size) + WDJS + PFM | 6 | 9724.774 | 27.299 |
| log(sizeNext) ~ log(size) + block + WDJS + PFM | 7 | 9725.234 | 27.759 |
| log(sizeNext) ~ log(size) + WDJS + PFM + TmDF | 7 | 9726.584 | 29.109 |
| log(sizeNext) ~ log(size) + block + WDJS + PFM + TmDF | 8 | 9727.023 | 29.548 |
| log(sizeNext) ~ log(size) + Intra + WDJS + TmDF | 7 | 9758.658 | 61.183 |
| log(sizeNext) ~ log(size) + Intra + block + WDJS + TmDF | 8 | 9760.364 | 62.889 |
| log(sizeNext) ~ log(size) + Intra + Inter + WDJS + TmDF | 8 | 9760.367 | 62.892 |
| log(sizeNext) ~ log(size) + Intra + Inter + block + WDJS + TmDF | 9 | 9762.110 | 64.635 |
| log(sizeNext) ~ log(size) + WDJS + TmDF | 6 | 9774.916 | 77.441 |
| log(sizeNext) ~ log(size) + block + WDJS + TmDF | 7 | 9775.604 | 78.130 |
| log(sizeNext) ~ log(size) + Inter + WDJS + TmDF | 7 | 9776.198 | 78.723 |
| log(sizeNext) ~ log(size) + Inter + block + WDJS + TmDF | 8 | 9777.022 | 79.547 |
| log(sizeNext) ~ log(size) + Intra + WDJS | 6 | 9839.314 | 141.839 |
| log(sizeNext) ~ log(size) + Intra + Inter + WDJS | 7 | 9839.735 | 142.261 |
| log(sizeNext) ~ log(size) + Intra + block + WDJS | 7 | 9841.072 | 143.597 |
| log(sizeNext) ~ log(size) + Intra + Inter + block + WDJS | 8 | 9841.565 | 144.090 |
| log(sizeNext) ~ log(size) + Inter + WDJS | 6 | 9867.962 | 170.487 |
| log(sizeNext) ~ log(size) + Inter + block + WDJS | 7 | 9868.615 | 171.141 |
| log(sizeNext) ~ log(size) + WDJS | 5 | 9868.881 | 171.406 |
| log(sizeNext) ~ log(size) + block + WDJS | 6 | 9869.225 | 171.750 |
| log(sizeNext) ~ log(size) + Intra + Inter + PFM + TmDF | 8 | 9870.927 | 173.452 |
| log(sizeNext) ~ log(size) + Intra + Inter + block + PFM + TmDF | 9 | 9872.531 | 175.056 |
| log(sizeNext) ~ log(size) + Intra + PFM + TmDF | 7 | 9881.051 | 183.576 |
| log(sizeNext) ~ log(size) + Intra + block + PFM + TmDF | 8 | 9882.305 | 184.831 |
| log(sizeNext) ~ log(size) + Intra + Inter + PFM | 7 | 9885.936 | 188.462 |
| log(sizeNext) ~ log(size) + Intra + Inter + block + PFM | 8 | 9887.399 | 189.924 |
| log(sizeNext) ~ log(size) + Intra + PFM | 6 | 9890.052 | 192.577 |
| log(sizeNext) ~ log(size) + Inter + PFM + TmDF | 7 | 9890.555 | 193.080 |
| log(sizeNext) ~ log(size) + Inter + block + PFM + TmDF | 8 | 9891.011 | 193.536 |
| log(sizeNext) ~ log(size) + Intra + block + PFM | 7 | 9891.265 | 193.791 |
| log(sizeNext) ~ log(size) + Inter + PFM | 6 | 9900.270 | 202.795 |
| log(sizeNext) ~ log(size) + Inter + block + PFM | 7 | 9900.684 | 203.210 |
| log(sizeNext) ~ log(size) + block + PFM + TmDF | 7 | 9901.579 | 204.105 |
| log(sizeNext) ~ log(size) + PFM + TmDF | 6 | 9901.862 | 204.388 |
| log(sizeNext) ~ log(size) + block + PFM | 6 | 9906.067 | 208.592 |
| log(sizeNext) ~ log(size) + PFM | 5 | 9906.220 | 208.745 |
| log(sizeNext) ~ log(size) + Intra + TmDF | 6 | 10008.384 | 310.910 |
| log(sizeNext) ~ log(size) + Intra + block + TmDF | 7 | 10009.517 | 312.042 |
| log(sizeNext) ~ log(size) + Intra + Inter + TmDF | 7 | 10010.291 | 312.816 |
| log(sizeNext) ~ log(size) + Intra + Inter + block + TmDF | 8 | 10011.457 | 313.982 |
| log(sizeNext) ~ log(size) + TmDF | 5 | 10018.290 | 320.815 |
| log(sizeNext) ~ log(size) + block + TmDF | 6 | 10018.342 | 320.867 |
| log(sizeNext) ~ log(size) + Inter + TmDF | 6 | 10019.978 | 322.503 |
| log(sizeNext) ~ log(size) + Inter + block + TmDF | 7 | 10020.129 | 322.654 |
| log(sizeNext) ~ log(size) + Intra | 5 | 10128.078 | 430.603 |
| log(sizeNext) ~ log(size) + Intra + Inter | 6 | 10128.700 | 431.225 |
| log(sizeNext) ~ log(size) + Intra + block | 6 | 10129.276 | 431.802 |
| log(sizeNext) ~ log(size) + Intra + Inter + block | 7 | 10130.015 | 432.541 |
| log(sizeNext) ~ log(size) + Inter + block | 6 | 10150.628 | 453.153 |
| log(sizeNext) ~ log(size) + block | 5 | 10150.788 | 453.313 |
| log(sizeNext) ~ log(size) + Inter | 5 | 10150.789 | 453.314 |
| log(sizeNext) ~ log(size) | 4 | 10151.278 | 453.803 |
| log(sizeNext) ~ 1 | 3 | 13450.399 | 3752.924 |

**Table S3.** AIC table of GLMMs for *H. squamatum* probability of reproduction. All models were adjusted with the function *glmer()* in package *lme4* (Bates et al. 2014), including plot as random factor and using binomial error distribution. K: number of estimated parameters for each model; AICc: Akaike weights. Abbreviations for explanatory variables: WDJS: water deficit from June to September; PFM: rainfall from February to May; TmDF: average minimum temperature from December to February; Intra: intraspecific interaction index; Inter: interspecific interaction index. Selected model is highlighted in bold.

| Model | K | AICc | ΔAICc |
| --- | --- | --- | --- |
| **Rep ~ log(size) + Intra + Inter + block + PFM + TmDF** | **8** | **3717.604** | **0.000** |
| Rep ~ log(size) + Intra + block + PFM + TmDF | 7 | 3718.636 | 1.031 |
| Rep ~ log(size) + Intra + Inter + PFM + TmDF | 7 | 3726.671 | 9.067 |
| Rep ~ log(size) + Intra + PFM + TmDF | 6 | 3726.937 | 9.332 |
| Rep ~ log(size) + Inter + block + PFM + TmDF | 7 | 3728.814 | 11.210 |
| Rep ~ log(size) + block + PFM + TmDF | 6 | 3730.604 | 13.000 |
| Rep ~ log(size) + Inter + PFM + TmDF | 6 | 3735.882 | 18.277 |
| Rep ~ log(size) + PFM + TmDF | 5 | 3736.822 | 19.217 |
| Rep ~ log(size) + Inter + block + PFM | 6 | 4348.158 | 630.554 |
| Rep ~ log(size) + Intra + Inter + block + PFM | 7 | 4348.238 | 630.634 |
| Rep ~ log(size) + Inter + PFM | 5 | 4358.121 | 640.516 |
| Rep ~ log(size) + Intra + Inter + PFM | 6 | 4359.357 | 641.753 |
| Rep ~ log(size) + block + PFM | 5 | 4363.460 | 645.855 |
| Rep ~ log(size) + Intra + block + PFM | 6 | 4364.216 | 646.612 |
| Rep ~ log(size) + PFM | 4 | 4377.587 | 659.983 |
| Rep ~ log(size) + Intra + PFM | 5 | 4379.370 | 661.766 |
| Rep ~ log(size) + Intra + Inter + block + TmDF | 7 | 4463.369 | 745.765 |
| Rep ~ log(size) + Inter + block + TmDF | 6 | 4465.019 | 747.415 |
| Rep ~ log(size) + Intra + Inter + TmDF | 6 | 4471.422 | 753.818 |
| Rep ~ log(size) + Inter + TmDF | 5 | 4471.721 | 754.117 |
| Rep ~ log(size) + Intra + Inter + block | 6 | 4475.796 | 758.191 |
| Rep ~ log(size) + Inter + block | 5 | 4476.586 | 758.982 |
| Rep ~ log(size) + Intra + block + TmDF | 6 | 4480.508 | 762.904 |
| Rep ~ log(size) + block + TmDF | 5 | 4481.206 | 763.601 |
| Rep ~ log(size) + Inter | 4 | 4484.057 | 766.453 |
| Rep ~ log(size) + Intra + Inter | 5 | 4484.530 | 766.925 |
| Rep ~ log(size) + TmDF | 4 | 4491.410 | 773.806 |
| Rep ~ log(size) + Intra + TmDF | 5 | 4492.101 | 774.497 |
| Rep ~ log(size) + block | 4 | 4496.172 | 778.568 |
| Rep ~ log(size) + Intra + block | 5 | 4496.318 | 778.714 |
| Rep ~ log(size) | 3 | 4507.936 | 790.331 |
| Rep ~ log(size) + Intra | 4 | 4509.309 | 791.704 |
| Rep ~ 1 | 2 | 6934.404 | 3216.799 |

**Table S4.** AIC table of GLMMs for *H. squamatum* fecundity. All models were adjusted with the function *glmer()* in package *lme4* (Bates et al. 2014), including plot as random factor and using Poisson error distribution. K: number of estimated parameters for each model; AICc: Akaike weights. Abbreviations for explanatory variables: WDJS: water deficit from June to September; PFM: rainfall from February to May; TmDF: average minimum temperature from December to February; Intra: intraspecific interaction index; Inter: interspecific interaction index. Selected model is highlighted in bold.

| Model | K | AICc | ΔAICc |
| --- | --- | --- | --- |
| **Fec ~ log(size) + Intra + Inter + block + WDJS + PFM + TmDF** | **9** | **9421.443** | **0.000** |
| fec ~ log(size) + Intra + block + WDJS + PFM + TmDF | 8 | 9421.673 | 0.230 |
| fec ~ log(size) + Intra + Inter + WDJS + PFM + TmDF | 8 | 9424.394 | 2.951 |
| fec ~ log(size) + Intra + WDJS + PFM + TmDF | 7 | 9424.544 | 3.101 |
| fec ~ log(size) + Intra + Inter + block + PFM + TmDF | 8 | 10042.377 | 620.934 |
| fec ~ log(size) + Intra + block + PFM + TmDF | 7 | 10043.798 | 622.355 |
| fec ~ log(size) + Intra + Inter + PFM + TmDF | 7 | 10044.801 | 623.358 |
| fec ~ log(size) + Intra + PFM + TmDF | 6 | 10046.322 | 624.878 |
| fec ~ log(size) + Inter + WDJS + PFM + TmDF | 7 | 10183.541 | 762.098 |
| fec ~ log(size) + WDJS + PFM + TmDF | 6 | 10184.070 | 762.627 |
| fec ~ log(size) + Inter + block + WDJS + PFM + TmDF | 8 | 10184.270 | 762.827 |
| fec ~ log(size) + block + WDJS + PFM + TmDF | 7 | 10184.846 | 763.403 |
| fec ~ log(size) + PFM + TmDF | 5 | 10588.543 | 1167.100 |
| fec ~ log(size) + block + PFM + TmDF | 6 | 10589.019 | 1167.576 |
| fec ~ log(size) + Inter + PFM + TmDF | 6 | 10589.715 | 1168.272 |
| fec ~ log(size) + Inter + block + PFM + TmDF | 7 | 10590.225 | 1168.782 |
| fec ~ log(size) + Intra + Inter + block + WDJS + PFM | 8 | 11895.684 | 2474.241 |
| fec ~ log(size) + Intra + Inter + WDJS + PFM | 7 | 11898.822 | 2477.379 |
| fec ~ log(size) + Intra + block + WDJS + PFM | 7 | 12133.319 | 2711.876 |
| fec ~ log(size) + Intra + WDJS + PFM | 6 | 12135.694 | 2714.251 |
| fec ~ log(size) + Intra + Inter + block + WDJS + TmDF | 8 | 12188.377 | 2766.934 |
| fec ~ log(size) + Intra + Inter + WDJS + TmDF | 7 | 12191.324 | 2769.881 |
| fec ~ log(size) + Intra + Inter + block + WDJS | 7 | 12333.575 | 2912.132 |
| fec ~ log(size) + Intra + Inter + WDJS | 6 | 12336.563 | 2915.120 |
| fec ~ log(size) + Intra + Inter + block + PFM | 7 | 12390.230 | 2968.787 |
| fec ~ log(size) + Intra + Inter + PFM | 6 | 12393.322 | 2971.879 |
| fec ~ log(size) + Intra + block + WDJS + TmDF | 7 | 12501.120 | 3079.677 |
| fec ~ log(size) + Intra + WDJS + TmDF | 6 | 12503.065 | 3081.622 |
| fec ~ log(size) + Intra + block + PFM | 6 | 12562.759 | 3141.316 |
| fec ~ log(size) + Intra + PFM | 5 | 12565.201 | 3143.758 |
| fec ~ log(size) + Intra + block + WDJS | 6 | 12683.763 | 3262.320 |
| fec ~ log(size) + Intra + WDJS | 5 | 12685.730 | 3264.287 |
| fec ~ log(size) + Intra + Inter + block + TmDF | 7 | 13018.549 | 3597.105 |
| fec ~ log(size) + Intra + Inter + TmDF | 6 | 13021.052 | 3599.609 |
| fec ~ log(size) + Inter + WDJS + PFM | 6 | 13064.421 | 3642.978 |
| fec ~ log(size) + Inter + block + WDJS + PFM | 7 | 13065.114 | 3643.671 |
| fec ~ log(size) + Intra + Inter + block | 6 | 13067.980 | 3646.537 |
| fec ~ log(size) + Intra + Inter | 5 | 13070.620 | 3649.176 |
| fec ~ log(size) + Intra + block + TmDF | 6 | 13294.388 | 3872.945 |
| fec ~ log(size) + Intra + TmDF | 5 | 13295.930 | 3874.487 |
| fec ~ log(size) + Inter + WDJS + TmDF | 6 | 13353.145 | 3931.702 |
| fec ~ log(size) + Inter + block + WDJS + TmDF | 7 | 13353.883 | 3932.440 |
| fec ~ log(size) + Intra + block | 5 | 13367.092 | 3945.649 |
| fec ~ log(size) + Intra | 4 | 13368.769 | 3947.326 |
| fec ~ log(size) + Inter + PFM | 5 | 13388.657 | 3967.214 |
| fec ~ log(size) + WDJS + PFM | 5 | 13388.988 | 3967.545 |
| fec ~ log(size) + Inter + block + PFM | 6 | 13389.074 | 3967.631 |
| fec ~ log(size) + block + WDJS + PFM | 6 | 13390.270 | 3968.826 |
| fec ~ log(size) + Inter + WDJS | 5 | 13531.189 | 4109.745 |
| fec ~ log(size) + Inter + block + WDJS | 6 | 13531.907 | 4110.464 |
| fec ~ log(size) + PFM | 4 | 13661.220 | 4239.777 |
| fec ~ log(size) + block + PFM | 5 | 13662.251 | 4240.807 |
| fec ~ log(size) + WDJS + TmDF | 5 | 13734.300 | 4312.857 |
| fec ~ log(size) + block + WDJS + TmDF | 6 | 13735.735 | 4314.292 |
| fec ~ log(size) + Inter + TmDF | 5 | 13933.952 | 4512.509 |
| fec ~ log(size) + Inter + block + TmDF | 6 | 13934.501 | 4513.058 |
| fec ~ log(size) + WDJS | 4 | 13970.125 | 4548.682 |
| fec ~ log(size) + block + WDJS | 5 | 13971.571 | 4550.128 |
| fec ~ log(size) + Inter | 4 | 14031.329 | 4609.886 |
| fec ~ log(size) + Inter + block | 5 | 14031.825 | 4610.382 |
| fec ~ log(size) + TmDF | 4 | 14291.670 | 4870.227 |
| fec ~ log(size) + block + TmDF | 5 | 14292.969 | 4871.526 |
| fec ~ log(size) | 3 | 14433.139 | 5011.696 |
| fec ~ log(size) + block | 4 | 14434.417 | 5012.974 |
| fec ~ 1 | 2 | 20445.898 | 11024.455 |

**Table S5.** AIC table of GLMMs for *L. subulatum* survival. All models were adjusted with the function *glmer()* in package *lme4* (Bates et al. 2014), including plot as random factor and using binomial error distribution. K: number of estimated parameters for each model; AICc: Akaike weights. Abbreviations for explanatory variables: WDJS: water deficit from June to September; PFM: rainfall from February to May; TmDF: average minimum temperature from December to February; Intra: intraspecific interaction index; Inter: interspecific interaction index. Selected model is highlighted in bold.

| Model | K | AICc | ΔAICc |
| --- | --- | --- | --- |
| Surv ~ log(size) + Inter + WDJS + PFM + TmDF | 7 | 1230.304 | 0.000 |
| **Surv ~ log(size) + Intra + Inter + WDJS + PFM + TmDF** | **8** | **1230.982** | **0.678** |
| Surv ~ log(size) + Inter + WDJS + TmDF | 6 | 1231.670 | 1.365 |
| Surv ~ log(size) + Intra + Inter + WDJS + TmDF | 7 | 1231.830 | 1.525 |
| Surv ~ log(size) + Inter + block + WDJS + PFM + TmDF | 8 | 1232.215 | 1.910 |
| Surv ~ log(size) + Intra + Inter + block + WDJS + PFM + TmDF | 9 | 1232.858 | 2.554 |
| Surv ~ log(size) + Inter + block + WDJS + TmDF | 7 | 1233.517 | 3.212 |
| Surv ~ log(size) + Intra + Inter + block + WDJS + TmDF | 8 | 1233.595 | 3.291 |
| Surv ~ log(size) + WDJS + PFM + TmDF | 6 | 1239.320 | 9.015 |
| Surv ~ log(size) + Intra + WDJS + PFM + TmDF | 7 | 1240.142 | 9.838 |
| Surv ~ log(size) + block + WDJS + PFM + TmDF | 7 | 1241.339 | 11.035 |
| Surv ~ log(size) + WDJS + TmDF | 5 | 1241.612 | 11.308 |
| Surv ~ log(size) + Intra + WDJS + TmDF | 6 | 1241.835 | 11.531 |
| Surv ~ log(size) + Intra + Inter + WDJS + PFM | 7 | 1241.931 | 11.626 |
| Surv ~ log(size) + Intra + block + WDJS + PFM + TmDF | 8 | 1242.157 | 11.852 |
| Surv ~ log(size) + Inter + WDJS + PFM | 6 | 1242.315 | 12.011 |
| Surv ~ log(size) + Intra + Inter + block + WDJS + PFM | 8 | 1243.528 | 13.224 |
| Surv ~ log(size) + block + WDJS + TmDF | 6 | 1243.610 | 13.306 |
| Surv ~ log(size) + Intra + block + WDJS + TmDF | 7 | 1243.801 | 13.497 |
| Surv ~ log(size) + Inter + block + WDJS + PFM | 7 | 1244.058 | 13.754 |
| Surv ~ log(size) + Inter + WDJS | 5 | 1245.097 | 14.793 |
| Surv ~ log(size) + Intra + Inter + WDJS | 6 | 1245.127 | 14.823 |
| Surv ~ log(size) + Intra + Inter + block + WDJS | 7 | 1246.769 | 16.465 |
| Surv ~ log(size) + Inter + block + WDJS | 6 | 1246.841 | 16.537 |
| Surv ~ log(size) + Intra + WDJS + PFM | 6 | 1254.453 | 24.149 |
| Surv ~ log(size) + WDJS + PFM | 5 | 1254.812 | 24.507 |
| Surv ~ log(size) + Intra + block + WDJS + PFM | 7 | 1256.335 | 26.030 |
| Surv ~ log(size) + block + WDJS + PFM | 6 | 1256.769 | 26.465 |
| Surv ~ log(size) + WDJS | 4 | 1258.588 | 28.283 |
| Surv ~ log(size) + Intra + WDJS | 5 | 1258.734 | 28.429 |
| Surv ~ log(size) + block + WDJS | 5 | 1260.546 | 30.240 |
| Surv ~ log(size) + Intra + block + WDJS | 6 | 1260.646 | 30.341 |
| Surv ~ log(size) + Intra + Inter + PFM + TmDF | 7 | 1316.500 | 86.195 |
| Surv ~ log(size) + Inter + PFM + TmDF | 6 | 1316.634 | 86.329 |
| Surv ~ log(size) + Intra + Inter + block + PFM + TmDF | 8 | 1318.526 | 88.221 |
| Surv ~ log(size) + Inter + block + PFM + TmDF | 7 | 1318.659 | 88.354 |
| Surv ~ log(size) + PFM + TmDF | 5 | 1330.780 | 100.476 |
| Surv ~ log(size) + Intra + PFM + TmDF | 6 | 1330.863 | 100.559 |
| Surv ~ log(size) + block + PFM + TmDF | 6 | 1332.695 | 102.390 |
| Surv ~ log(size) + Intra + block + PFM + TmDF | 7 | 1332.762 | 102.457 |
| Surv ~ log(size) + Intra + Inter + TmDF | 6 | 1368.165 | 137.860 |
| Surv ~ log(size) + Intra + Inter + block + TmDF | 7 | 1370.158 | 139.853 |
| Surv ~ log(size) + Inter + TmDF | 5 | 1371.234 | 140.929 |
| Surv ~ log(size) + Inter + block + TmDF | 6 | 1373.254 | 142.950 |
| Surv ~ log(size) + Intra + Inter + PFM | 6 | 1374.607 | 144.302 |
| Surv ~ log(size) + Intra + Inter | 5 | 1375.883 | 145.579 |
| Surv ~ log(size) + Intra + Inter + block + PFM | 7 | 1376.546 | 146.241 |
| Surv ~ log(size) + Inter + PFM | 5 | 1377.265 | 146.960 |
| Surv ~ log(size) + Intra + Inter + block | 6 | 1377.824 | 147.520 |
| Surv ~ log(size) + Inter | 4 | 1379.070 | 148.766 |
| Surv ~ log(size) + Inter + block + PFM | 6 | 1379.258 | 148.953 |
| Surv ~ log(size) + Inter + block | 5 | 1381.070 | 150.766 |
| Surv ~ log(size) + Intra + TmDF | 5 | 1386.512 | 156.208 |
| Surv ~ log(size) + Intra + block + TmDF | 6 | 1388.531 | 158.226 |
| Surv ~ log(size) + TmDF | 4 | 1389.608 | 159.303 |
| Surv ~ log(size) + block + TmDF | 5 | 1391.574 | 161.270 |
| Surv ~ log(size) + Intra + PFM | 5 | 1396.197 | 165.892 |
| Surv ~ log(size) + Intra | 4 | 1396.875 | 166.571 |
| Surv ~ log(size) + Intra + block + PFM | 6 | 1398.219 | 167.914 |
| Surv ~ log(size) + PFM | 4 | 1398.858 | 168.553 |
| Surv ~ log(size) + Intra + block | 5 | 1398.893 | 168.589 |
| Surv ~ log(size) | 3 | 1400.052 | 169.748 |
| Surv ~ log(size) + block + PFM | 5 | 1400.858 | 170.554 |
| Surv ~ log(size) + block | 4 | 1402.045 | 171.741 |
| Surv ~ 1 | 2 | 1450.283 | 219.979 |

**Table S6.** AIC table of GLMMs for *L. subulatum* growth. All models were adjusted with the function *lme()* in package *nlme* (Pinheiro et al. 2015), including plot as random factor and using Gaussian error distribution. K: number of estimated parameters for each model; AICc: Akaike weights. Abbreviations for explanatory variables: WDJS: water deficit from June to September; PFM: rainfall from February to May; TmDF: average minimum temperature from December to February; Intra: intraspecific interaction index; Inter: interspecific interaction index. Selected model is highlighted in bold.

| Model | K | AICc | ΔAICc |
| --- | --- | --- | --- |
| log(sizeNext) ~ log(size) + WDJS + PFM + TmDF | 7 | 1840.303 | 0.000 |
| **log(sizeNext) ~ log(size) + Inter + WDJS + PFM + TmDF** | **8** | **1840.970** | **0.667** |
| log(sizeNext) ~ log(size) + Intra + WDJS + PFM + TmDF | 8 | 1842.134 | 1.831 |
| log(sizeNext) ~ log(size) + block + WDJS + PFM + TmDF | 8 | 1842.335 | 2.032 |
| log(sizeNext) ~ log(size) + Intra + Inter + WDJS + PFM + TmDF | 9 | 1842.838 | 2.534 |
| log(sizeNext) ~ log(size) + Inter + block + WDJS + PFM + TmDF | 9 | 1843.022 | 2.719 |
| log(sizeNext) ~ log(size) + Intra + block + WDJS + PFM + TmDF | 9 | 1844.147 | 3.844 |
| log(sizeNext) ~ log(size) + Intra + Inter + block + WDJS + PFM + TmDF | 10 | 1844.886 | 4.583 |
| log(sizeNext) ~ log(size) + WDJS + PFM | 6 | 1901.083 | 60.780 |
| log(sizeNext) ~ log(size) + Intra + WDJS + PFM | 7 | 1901.110 | 60.807 |
| log(sizeNext) ~ log(size) + Intra + block + WDJS + PFM | 8 | 1902.184 | 61.880 |
| log(sizeNext) ~ log(size) + block + WDJS + PFM | 7 | 1902.508 | 62.205 |
| log(sizeNext) ~ log(size) + Inter + WDJS + PFM | 7 | 1902.981 | 62.678 |
| log(sizeNext) ~ log(size) + Intra + Inter + WDJS + PFM | 8 | 1903.041 | 62.738 |
| log(sizeNext) ~ log(size) + Intra + Inter + block + WDJS + PFM | 9 | 1904.155 | 63.852 |
| log(sizeNext) ~ log(size) + Inter + block + WDJS + PFM | 8 | 1904.442 | 64.138 |
| log(sizeNext) ~ log(size) + Intra + WDJS + TmDF | 7 | 1911.627 | 71.323 |
| log(sizeNext) ~ log(size) + Intra + WDJS | 6 | 1911.845 | 71.541 |
| log(sizeNext) ~ log(size) + Intra + block + WDJS + TmDF | 8 | 1912.520 | 72.217 |
| log(sizeNext) ~ log(size) + Intra + block + WDJS | 7 | 1912.651 | 72.348 |
| log(sizeNext) ~ log(size) + WDJS + TmDF | 6 | 1913.326 | 73.023 |
| log(sizeNext) ~ log(size) + WDJS | 5 | 1913.370 | 73.066 |
| log(sizeNext) ~ log(size) + Intra + Inter + WDJS + TmDF | 8 | 1913.533 | 73.229 |
| log(sizeNext) ~ log(size) + Intra + Inter + WDJS | 7 | 1913.800 | 73.497 |
| log(sizeNext) ~ log(size) + Intra + Inter + block + WDJS + TmDF | 9 | 1914.474 | 74.170 |
| log(sizeNext) ~ log(size) + Intra + Inter + block + WDJS | 8 | 1914.644 | 74.341 |
| log(sizeNext) ~ log(size) + block + WDJS | 6 | 1914.658 | 74.355 |
| log(sizeNext) ~ log(size) + block + WDJS + TmDF | 7 | 1914.688 | 74.385 |
| log(sizeNext) ~ log(size) + Inter + WDJS + TmDF | 7 | 1915.179 | 74.876 |
| log(sizeNext) ~ log(size) + Inter + WDJS | 6 | 1915.281 | 74.978 |
| log(sizeNext) ~ log(size) + Inter + block + WDJS + TmDF | 8 | 1916.583 | 76.279 |
| log(sizeNext) ~ log(size) + Inter + block + WDJS | 7 | 1916.605 | 76.301 |
| log(sizeNext) ~ log(size) + PFM + TmDF | 6 | 2023.456 | 183.150 |
| log(sizeNext) ~ log(size) + Inter + PFM + TmDF | 7 | 2023.570 | 183.266 |
| log(sizeNext) ~ log(size) + block + PFM + TmDF | 7 | 2025.289 | 184.986 |
| log(sizeNext) ~ log(size) + Inter + block + PFM + TmDF | 8 | 2025.297 | 184.994 |
| log(sizeNext) ~ log(size) + Intra + PFM + TmDF | 7 | 2025.498 | 185.195 |
| log(sizeNext) ~ log(size) + Intra + Inter + PFM + TmDF | 8 | 2025.617 | 185.314 |
| log(sizeNext) ~ log(size) + Intra + block + PFM + TmDF | 8 | 2027.333 | 187.030 |
| log(sizeNext) ~ log(size) + Intra + Inter + block + PFM + TmDF | 9 | 2027.339 | 187.036 |
| log(sizeNext) ~ log(size) + PFM | 5 | 2090.958 | 250.655 |
| log(sizeNext) ~ log(size) + Intra + PFM | 6 | 2091.944 | 251.640 |
| log(sizeNext) ~ log(size) + Inter + PFM | 6 | 2092.751 | 252.448 |
| log(sizeNext) ~ log(size) + block + PFM | 6 | 2092.959 | 252.656 |
| log(sizeNext) ~ log(size) + Intra + Inter + PFM | 7 | 2093.757 | 253.454 |
| log(sizeNext) ~ log(size) + Intra + block + PFM | 7 | 2093.880 | 253.577 |
| log(sizeNext) ~ log(size) + Inter + block + PFM | 7 | 2094.768 | 254.465 |
| log(sizeNext) ~ log(size) + Intra + Inter + block + PFM | 8 | 2095.717 | 255.414 |
| log(sizeNext) ~ log(size) + Intra | 5 | 2137.883 | 297.580 |
| log(sizeNext) ~ log(size) + Intra + TmDF | 6 | 2138.739 | 298.435 |
| log(sizeNext) ~ log(size) | 4 | 2139.582 | 299.279 |
| log(sizeNext) ~ log(size) + Intra + block | 6 | 2139.669 | 299.366 |
| log(sizeNext) ~ log(size) + Intra + Inter | 6 | 2139.765 | 299.461 |
| log(sizeNext) ~ log(size) + TmDF | 5 | 2140.275 | 299.972 |
| log(sizeNext) ~ log(size) + Intra + block + TmDF | 7 | 2140.498 | 300.195 |
| log(sizeNext) ~ log(size) + Intra + Inter + TmDF | 7 | 2140.673 | 300.370 |
| log(sizeNext) ~ log(size) + Inter | 5 | 2141.425 | 301.122 |
| log(sizeNext) ~ log(size) + block | 5 | 2141.546 | 301.243 |
| log(sizeNext) ~ log(size) + Intra + Inter + block | 7 | 2141.577 | 301.274 |
| log(sizeNext) ~ log(size) + Inter + TmDF | 6 | 2142.181 | 301.878 |
| log(sizeNext) ~ log(size) + block + TmDF | 6 | 2142.225 | 301.921 |
| log(sizeNext) ~ log(size) + Intra + Inter + block + TmDF | 8 | 2142.457 | 302.154 |
| log(sizeNext) ~ log(size) + Inter + block | 6 | 2143.407 | 303.104 |
| log(sizeNext) ~ log(size) + Inter + block + TmDF | 7 | 2144.148 | 303.845 |
| log(sizeNext) ~ 1 | 3 | 3193.960 | 1353.657 |

**Table S7.** AIC table of GLMMs for *L. subulatum* probability of reproduction. All models were adjusted with the function *glmer()* in package *lme4* (Bates et al. 2014), including plot as random factor and using binomial error distribution. K: number of estimated parameters for each model; AICc: Akaike weights. Abbreviations for explanatory variables: WDJS: water deficit from June to September; PFM: rainfall from February to May; TmDF: average minimum temperature from December to February; Intra: intraspecific interaction index; Inter: interspecific interaction index. Selected model is highlighted in bold.

| Model | K | AICc | ΔAICc |
| --- | --- | --- | --- |
| Rep ~ log(size) + Intra | 4 | 346.763 | 0.000 |
| Rep ~ log(size) + Intra + PFM | 5 | 348.002 | 1.239 |
| **Rep ~ log(size) + Intra + block** | **5** | **348.343** | **1.580** |
| Rep ~ log(size) + Intra + TmDF | 5 | 348.686 | 1.923 |
| Rep ~ log(size) + Intra + Inter | 5 | 348.774 | 2.011 |
| Rep ~ log(size) + Intra + block + PFM | 6 | 349.506 | 2.742 |
| Rep ~ log(size) + Intra + PFM + TmDF | 6 | 349.534 | 2.771 |
| Rep ~ log(size) + Intra + Inter + PFM | 6 | 350.021 | 3.258 |
| Rep ~ log(size) + Intra + block + TmDF | 6 | 350.214 | 3.451 |
| Rep ~ log(size) + Intra + Inter + block | 6 | 350.338 | 3.575 |
| Rep ~ log(size) + Intra + Inter + TmDF | 6 | 350.697 | 3.934 |
| Rep ~ log(size) + Intra + block + PFM + TmDF | 7 | 351.144 | 4.381 |
| Rep ~ log(size) + Intra + Inter + block + PFM | 7 | 351.509 | 4.746 |
| Rep ~ log(size) + Intra + Inter + PFM + TmDF | 7 | 351.562 | 4.799 |
| Rep ~ log(size) | 3 | 351.736 | 4.973 |
| Rep ~ log(size) + Intra + Inter + block + TmDF | 7 | 352.202 | 5.439 |
| Rep ~ log(size) + PFM | 4 | 352.363 | 5.600 |
| Rep ~ log(size) + Intra + Inter + block + PFM + TmDF | 8 | 353.171 | 6.408 |
| Rep ~ log(size) + PFM + TmDF | 5 | 353.258 | 6.495 |
| Rep ~ log(size) + TmDF | 4 | 353.636 | 6.873 |
| Rep ~ log(size) + block | 4 | 353.678 | 6.915 |
| Rep ~ log(size) + Inter | 4 | 353.751 | 6.988 |
| Rep ~ log(size) + block + PFM | 5 | 354.256 | 7.493 |
| Rep ~ log(size) + Inter + PFM | 5 | 354.383 | 7.620 |
| Rep ~ log(size) + block + PFM + TmDF | 6 | 355.220 | 8.457 |
| Rep ~ log(size) + Inter + PFM + TmDF | 6 | 355.266 | 8.503 |
| Rep ~ log(size) + block + TmDF | 5 | 355.558 | 8.795 |
| Rep ~ log(size) + Inter + TmDF | 5 | 355.654 | 8.891 |
| Rep ~ log(size) + Inter + block | 5 | 355.694 | 8.931 |
| Rep ~ log(size) + Inter + block + PFM | 6 | 356.277 | 9.514 |
| Rep ~ log(size) + Inter + block + PFM + TmDF | 7 | 357.240 | 10.477 |
| Rep ~ log(size) + Inter + block + TmDF | 6 | 357.573 | 10.810 |
| Rep ~ 1 | 2 | 932.518 | 585.755 |

**Table S8.** AIC table of GLMMs for *L. subulatum* fecundity. All models were adjusted with the function *glmer()* in package *lme4* (Bates et al. 2014), including plot as random factor and using Poisson error distribution. K: number of estimated parameters for each model; AICc: Akaike weights. Abbreviations for explanatory variables: WDJS: water deficit from June to September; PFM: rainfall from February to May; TmDF: average minimum temperature from December to February; Intra: intraspecific interaction index; Inter: interspecific interaction index. Selected model is highlighted in bold.

| Model | K | AICc | ΔAICc |
| --- | --- | --- | --- |
| Fec ~ log(size) + Intra + Inter + WDJS + PFM + TmDF | 8 | 1620.410 | 0.000 |
| Fec ~ log(size) + Intra + Inter + WDJS + TmDF | 7 | 1621.947 | 1.537 |
| **Fec ~ log(size) + Intra + Inter + block + WDJS + PFM + TmDF** | **9** | **1622.281** | **1.870** |
| Fec ~ log(size) + Intra + Inter + block + WDJS + TmDF | 8 | 1623.738 | 3.328 |
| Fec ~ log(size) + Intra + WDJS + TmDF | 6 | 1627.985 | 7.575 |
| Fec ~ log(size) + Intra + WDJS + PFM + TmDF | 7 | 1628.080 | 7.670 |
| Fec ~ log(size) + Inter + WDJS + PFM + TmDF | 7 | 1628.311 | 7.901 |
| Fec ~ log(size) + Intra + block + WDJS + TmDF | 7 | 1629.575 | 9.165 |
| Fec ~ log(size) + Intra + block + WDJS + PFM + TmDF | 8 | 1629.735 | 9.325 |
| Fec ~ log(size) + Inter + WDJS + TmDF | 6 | 1633.797 | 13.386 |
| Fec ~ log(size) + WDJS + PFM + TmDF | 6 | 1634.224 | 13.814 |
| Fec ~ log(size) + Inter + block + WDJS + TmDF | 7 | 1635.699 | 15.289 |
| Fec ~ log(size) + block + WDJS + PFM + TmDF | 7 | 1636.025 | 15.615 |
| Fec ~ log(size) + WDJS + TmDF | 5 | 1637.222 | 16.812 |
| Fec ~ log(size) + block + WDJS + TmDF | 6 | 1638.973 | 18.563 |
| Fec ~ log(size) + Intra + Inter + WDJS + PFM | 7 | 1674.207 | 53.797 |
| Fec ~ log(size) + Intra + Inter + block + WDJS + PFM | 8 | 1676.265 | 55.855 |
| Fec ~ log(size) + Inter + WDJS + PFM | 6 | 1677.081 | 56.671 |
| Fec ~ log(size) + Inter + block + WDJS + PFM | 7 | 1679.165 | 58.754 |
| Fec ~ log(size) + Intra + Inter + block + WDJS | 8 | 1684.148 | 63.738 |
| Fec ~ log(size) + Intra + WDJS + PFM | 6 | 1690.833 | 70.423 |
| Fec ~ log(size) + WDJS + PFM | 5 | 1691.629 | 71.219 |
| Fec ~ log(size) + Intra + block + WDJS + PFM | 7 | 1692.751 | 72.341 |
| Fec ~ log(size) + block + WDJS + PFM | 6 | 1693.591 | 73.181 |
| Fec ~ log(size) + Intra + Inter + WDJS | 6 | 1887.571 | 267.161 |
| Fec ~ log(size) + Intra + Inter + block + WDJS | 7 | 1889.397 | 268.987 |
| Fec ~ log(size) + Intra + WDJS | 5 | 1893.440 | 273.030 |
| Fec ~ log(size) + Intra + block + WDJS | 6 | 1895.116 | 274.705 |
| Fec ~ log(size) + Inter + WDJS | 5 | 1928.092 | 307.682 |
| Fec ~ log(size) + WDJS | 4 | 1929.195 | 308.785 |
| Fec ~ log(size) + Inter + block + WDJS | 6 | 1930.073 | 309.663 |
| Fec ~ log(size) + block + WDJS | 5 | 1931.095 | 310.685 |
| Fec ~ log(size) + Intra + Inter + PFM + TmDF | 7 | 2275.454 | 655.044 |
| Fec ~ log(size) + Intra + Inter + block + PFM + TmDF | 8 | 2275.885 | 655.475 |
| Fec ~ log(size) + Intra + Inter + TmDF | 6 | 2278.493 | 658.083 |
| Fec ~ log(size) + Intra + Inter + block + TmDF | 7 | 2278.578 | 658.168 |
| Fec ~ log(size) + Intra + PFM + TmDF | 6 | 2280.519 | 660.109 |
| Fec ~ log(size) + Intra + block + PFM + TmDF | 7 | 2281.161 | 660.751 |
| Fec ~ log(size) + Inter + PFM + TmDF | 6 | 2282.115 | 661.704 |
| Fec ~ log(size) + Inter + block + PFM + TmDF | 7 | 2282.863 | 662.453 |
| Fec ~ log(size) + Intra + Inter + PFM | 6 | 2285.917 | 665.507 |
| Fec ~ log(size) + Intra + Inter + block + PFM | 7 | 2286.770 | 666.360 |
| Fec ~ log(size) + Intra + TmDF | 5 | 2287.285 | 666.874 |
| Fec ~ log(size) + Intra + PFM | 5 | 2287.409 | 666.998 |
| Fec ~ log(size) + Intra + block + TmDF | 6 | 2287.554 | 667.144 |
| Fec ~ log(size) + Inter + TmDF | 5 | 2288.302 | 667.892 |
| Fec ~ log(size) + Intra + block + PFM | 6 | 2288.334 | 667.924 |
| Fec ~ log(size) + Inter + block + TmDF | 6 | 2288.709 | 668.299 |
| Fec ~ log(size) + PFM + TmDF | 5 | 2288.741 | 668.331 |
| Fec ~ log(size) + block + PFM + TmDF | 6 | 2289.710 | 669.300 |
| Fec ~ log(size) + Inter + PFM | 5 | 2291.730 | 671.320 |
| Fec ~ log(size) + Inter + block + PFM | 6 | 2292.823 | 672.412 |
| Fec ~ log(size) + PFM | 4 | 2294.505 | 674.094 |
| Fec ~ log(size) + block + PFM | 5 | 2295.686 | 675.276 |
| Fec ~ log(size) + TmDF | 4 | 2300.533 | 680.123 |
| Fec ~ log(size) + block + TmDF | 5 | 2301.157 | 680.747 |
| Fec ~ log(size) + Intra + Inter | 5 | 2365.666 | 745.256 |
| Fec ~ log(size) + Intra + Inter + block | 6 | 2365.914 | 745.503 |
| Fec ~ log(size) + Intra | 4 | 2371.154 | 750.744 |
| Fec ~ log(size) + Intra + block | 5 | 2371.521 | 751.111 |
| Fec ~ log(size) + Inter | 4 | 2393.257 | 772.847 |
| Fec ~ log(size) + Inter + block | 5 | 2393.945 | 773.535 |
| Fec ~ log(size) | 3 | 2403.173 | 782.763 |
| Fec ~ log(size) + block | 4 | 2404.001 | 783.590 |
| Fec ~ 1 | 2 | 2510.320 | 889.909 |

**
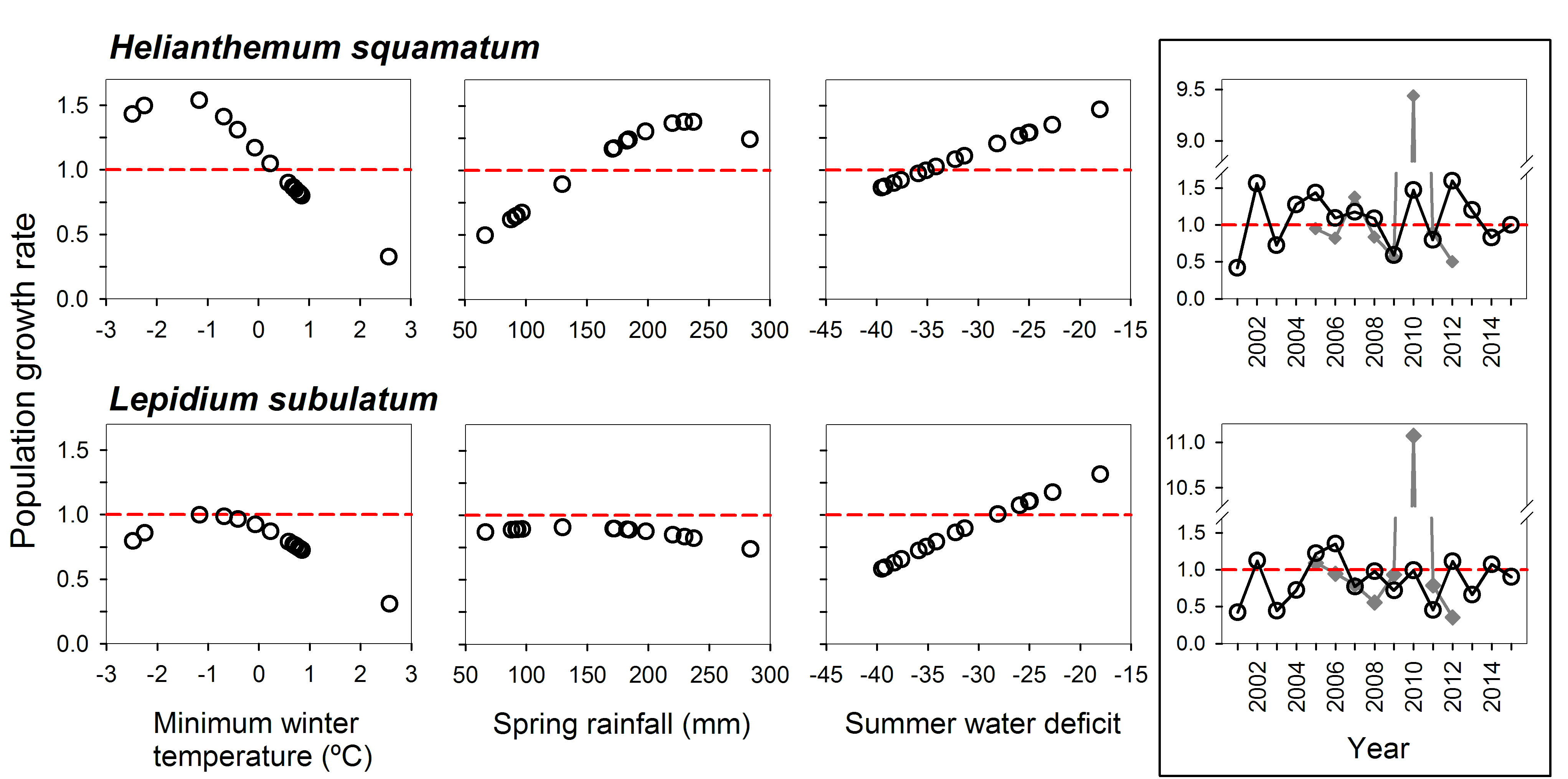
**

**Fig. S1.** Variation in population growth rate (lambda) with climatic variables (minimum winter temperature, spring rainfall and summer water balance) for *Helianthemum squamatum* and *Lepidium subulatum* at block B. See figure 2 for additional information.

**
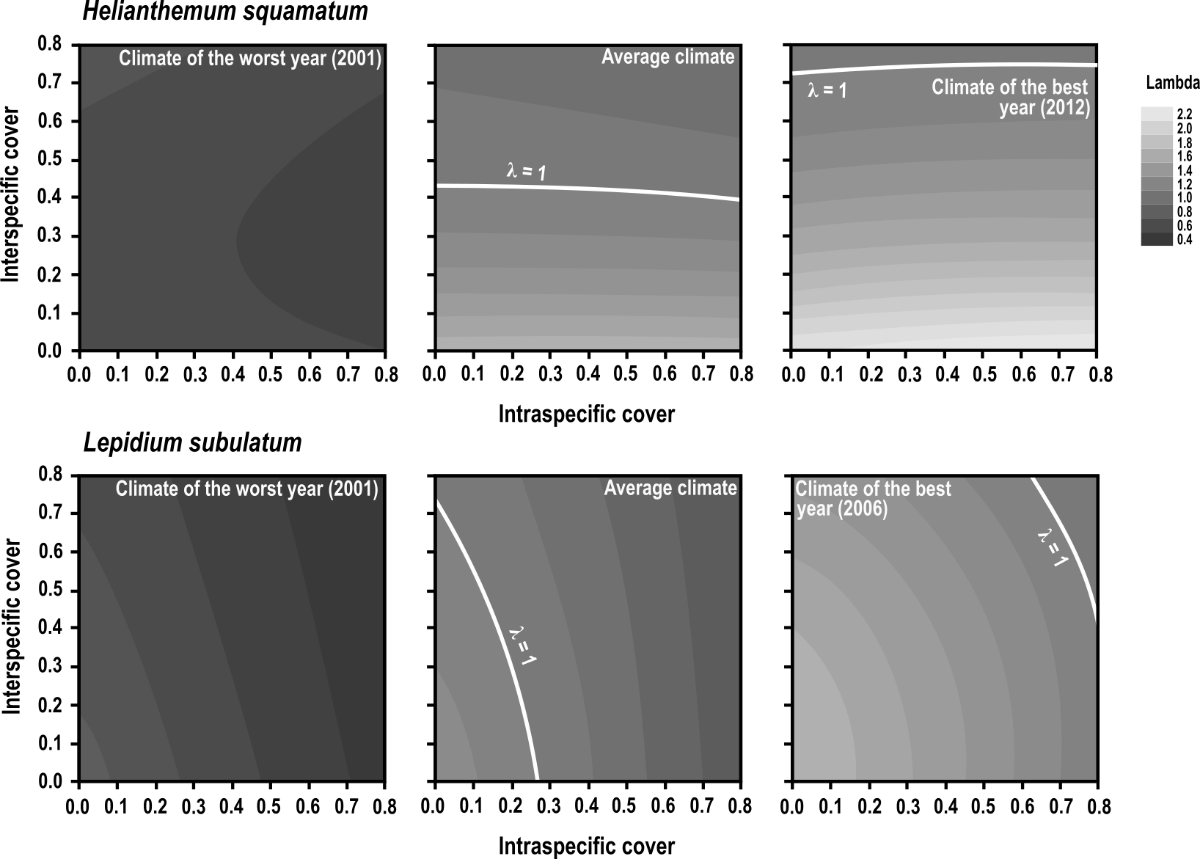
**

**Fig. S2.** Variation in population growth rate (lambda) depending on intra- and interspecific covers for *H. squamatum* and *L. subulatum* in block B. See figure 3 for additional information.

**Supplementary methods**

**Table S9.** Scenarios aimed at simulating variations in lambda under variable climate and recruitment intensity for 15 years (14 annual transitions).

| Climatic conditions ^a^ | Frequency ^b^ | Variation in recruitment ^c^ | Simulation No. |
| --- | --- | --- | --- |
| Variable | 1/15 | 1 | **1** |
|  | 1/15 | 2 | **2** |
|  | 1/15 | 5 | **3** |
|  | 1/15 | 10 | **4** |
| Conditions of 2001  (unfavourable for both species) | 1/3 | 1 | **5** |
|  | 1/3 | 2 | **6** |
|  | 1/3 | 5 | **7** |
|  | 1/3 | 10 | **8** |
| Conditions of 2012  (favourable for *H. squamatum*) | 1/3 | 1 | **9** |
|  | 1/3 | 2 | **10** |
|  | 1/3 | 5 | **11** |
|  | 1/3 | 10 | **12** |
| Conditions of 2006  (favourable for *L. subulatum*) | 1/4 | 1 | **13** |
|  | 1/4 | 2 | **14** |
|  | 1/4 | 5 | **15** |
|  | 1/4 | 10 | **16** |
| Conditions of 2012  and 2001 | 1/3 – 1/3 | 1 | **17** |
|  | 1/3 – 1/3 | 2 | **18** |
|  | 1/3 – 1/3 | 5 | **19** |
|  | 1/3 – 1/3 | 10 | **20** |
| Conditions of 2006  and 2001 | 1/4 – 1/3 | 1 | **21** |
|  | 1/4 – 1/3 | 2 | **22** |
|  | 1/4 – 1/3 | 5 | **23** |
|  | 1/4 – 1/3 | 10 | **24** |

^a^ Climatic data used for simulations correspond to observed climatic records for the period 2001-2015, shown in Table S10.

^b^ Under variable climate the frequency of occurrence is similar for all years (1/15). Frequency of 1/4 indicates that the probability of having climatic conditions of the year indicated in the first column (e.g. 2001 in the first case) is one in every four years, and accordingly for frequencies of 1/5 and 1/3.

^c^ Recruitment variation implies multiplying the number of seedlings by 1, 2, 5 or 10 fold when climatic conditions correspond with those of the climatically good year per species (2006 in *L. subulatum*, 2012 in *H. squamatum*).

Each scenario was replicated per block and considering either null or existing interaction between species.

Stochastic population growth rates of both species were recorded per scenario.

**Table S10.** Climatic data for the study period. Data were obtained from the Spanish Ministry of Agriculture, Food and Environment (http://crea.uclm.es/siar/datmeteo/), from the Barajas de Melo meteorological station (located 15 km away from the study area).

| Year | Spring rainfall ^a^ | Summer water balance ^b^ | Minimum winter temperature ^c^ |
| --- | --- | --- | --- |
| 2001 | 172.2 | -28.13 | 2.57 |
| 2002 | 171.2 | -22.72 | -0.41 |
| 2003 | 184.4 | -39.21 | 0.82 |
| 2004 | 283.8 | -32.26 | 0.59 |
| 2005 | 66.6 | -34.17 | -2.25 |
| 2006 | 87.8 | -25.95 | -0.68 |
| 2007 | 220.0 | -31.38 | 0.72 |
| 2008 | 182.6 | -25.07 | 0.85 |
| 2009 | 92.8 | -35.16 | -0.07 |
| 2010 | 237.4 | -18.03 | 0.78 |
| 2011 | 198.0 | -39.51 | 0.82 |
| 2012 | 91.6 | -35.90 | -2.48 |
| 2013 | 229.8 | -38.30 | 0.23 |
| 2014 | 129.8 | -24.96 | 0.68 |
| 2015 | 96.7 | -37.58 | -1.17 |

^a^ Accumulated rainfall from February to May

^b^ Water balance (*P – 2T*, where *P* is accumulated rainfall and *T* is mean temperature) from June to September

^c^ Averaged minimum temperature from December to February

**Methods M1.** Detailed description of the study species.

*Helianthemum squamatum* (Cistaceae) is a small, evergreen, drought-tolerant shrub that can live between 4 and 6 years (Olano, Eugenio & Escudero 2011), reproducing usually after the first year (Aragón *et al.* 2007; Quintana-Ascencio *et al.* 2009). Adults flower during late spring and summer, from May to August, and show an extremely high fecundity under a wide variety of environmental conditions (Aragón *et al.* 2007), although a close response to climate conditions is also present (Aragón, Escudero & Valladares 2008). Seeds adhere to the soil surface (Romão & Escudero 2005), incorporating to a dense, highly dynamic persistent, seed bank (Caballero *et al.* 2003, 2008; Olano, Caballero & Escudero 2012). Recruitment is associated with wet summers (Olano, Eugenio & Escudero 2011), and seedling survival is conditioned by factors operating at fine spatial and temporal scales, with complex interactions among them (Escudero *et al.* 1999, 2005; De la Cruz *et al.* 2008; Quintana-Ascencio *et al.* 2009).

*Lepidium subulatum* (Brassicaceae) is a small, summer-deciduous, drought-avoiding shrub that can live for more than 20 years and starts reproducing at 2-4 years (Soliveres *et al.* 2010; Eugenio *et al.* 2012). Adults flower in spring, from April to May, and fecundity shows high inter-annual variation. Seeds are small and have mucilage for anchoring close to the mother plant after dispersal (i.e., atelechory; Escudero *et al.* 2000), forming a small persistent seed bank (Caballero *et al.* 2008). Emergence is concentrated in winter, but can continue until as late as June (Escudero *et al.* 2000). Recruitment bottlenecks occur during the first summer after germination due to low survival rates (0.1 % to 10 %; Escudero *et al.* 2000), although survival increases sharply afterwards and reaches 71-95 % in adults. Differences between life cycles of both study species are summarized in Table S1.

**References**

Aragón, C. F., Albert, M. J., Giménez-Benavides, L., Luzuriaga, A. L. & Escudero, A. Environmental scales on the reproduction of a gypsophyte: A hierarchical approach. *Ann. Bot*. **99,** 519–527 (2007).

Aragón, C. F., Escudero, A. & Valladares, F. Stress-induced dynamic adjustments of reproduction differentially affect fitness components of a semiarid plant. *J. Ecol.* **96,** 222–229 (2008).

Caballero, I., Olano, J. M., Escudero, A. & Loidi, J. Seed bank structure along a semi-arid gypsum gradient in central Spain. *J. Arid Environ.* **55,** 287–299 (2003).

Caballero, I., Olano, J. M., Loidi, J. & Escudero, A. A model for small-scale seed bank and standing vegetation connection along time. *Oikos* **117,** 1788–1795 (2008).

De la Cruz, M., Romão, R. L., Escudero, A. & Maestre, F. T. Where do seedlings go? A spatio-temporal analysis of seedling mortality in a semi-arid gypsophyte. *Ecography* **31,** 720–730 (2008).

Escudero, A., Somolinos, R. C., Olano, J. M. & A. Rubio. Factors controlling the establishment of *Helianthemum squamatum* (L.) Dum., an endemic gypsophite of semi-arid Spain. *J. Ecol.* **87,** 290–302 (1999).

Escudero, A., Iriondo, J. M., Olano, J. M., Rubio, A. & Somolinos, R. Factors affecting establishment of a gypsophyte: the case of *Lepidium subulatum* (Brassicaceae). *Am. J. Bot.* **87**, 861–871 (2000).

Escudero, A., Romão, R., de la Cruz, M. & Maestre, F. T. Spatial pattern and neighbor effects on *Helianthemum squamatum* seedlings in a semiarid Mediterranean gypsum community. *J. Veg. Sci.* **16,** 383–390 (2005).

Eugenio, M., Olano, J. M., Ferrandis, P., Martínez-Duro, E.& Escudero, A. Population structure of two dominant gypsophyte shrubs through a secondary plant succession. *J. Arid Environ.* **76,** 30–35 (2012).

Olano, J. M., Eugenio, M. & Escudero, A. Site effect is stronger than species identity in driving demographic responses of *Helianthemum* (Cistaceae) shrubs in gypsum environments. *Am. J. Bot.* **98,** 1–8 (2011).

Olano, J. M., Caballero, I. & Escudero. A. Soil seed bank recovery occurs more rapidly than expected in semi-arid Mediterranean gypsum vegetation. *Ann. Bot.* **109,** 299–307 (2012).

Quintana-Ascencio, P. F., Caballero, I., Olano, J. M., Escudero, A. & Albert, M. J. Does habitat structure matter? Spatially explicit population modeling of an Iberian gypsum endemic. *Pop. Ecol.* **51,** 317–328 (2009).

Romão, R. L. & Escudero, A. Gypsum physical soil crusts and the existence of gypsophytes in semi-arid central Spain*. Plant Ecol.* **181,** 127–137 (2005).

Soliveres, S. L., DeSoto, L., Maestre, F. T. & Olano, J. M. Spatio-temporal heterogeneity in abiotic factors modulate multiple ontogenetic shifts between competition and facilitation. *Perspect. Plant Ecol. Evol. Syst.* **12,** 227–234 (2010).

**Table S11.** Comparison of different aspects of the life cycles of *H. squamatum* and *L. subulatum*. Superscripts indicate references: ^1^Olano, Eugenio & Escudero 2011, ^2^Aragón *et al.* 2007, ^3^Quintana-Ascencio *et al.* 2009, ^4^Olano, Caballero & Escudero 2012, ^5^Soliveres *et al.* 2010, ^6^Eugenio *et al.* 2012, ^7^Caballero *et al.* 2008, ^8^Escudero *et al.* 2000.

|  | *Helianthemum squamatum* | *Lepidium subulatum* |
| --- | --- | --- |
| **Strategy** | Drought tolerant | Drought avoider |
| **Leaf habit** | Evergreen | Summer deciduous, leaves present from September (October) to (May) June |
| **Flowering** | Low interannual variability, starts in May ^2^ | High interannual variability, starts in April |
| **Seed bank** | Dense and highly dynamic ^4^ | Small and very variable ^7^ |
| **Seedling emergence** | (Jan-Feb) March-April (May-June) ^8^ | |
| **Seedling survivorship** | High mortality (even 100%) at first year caused by summer drought ^1,8^ | |
| **Seedling growth** | Very fast | Very slow (at least aboveground) |
| **Age at maturity** | 1 year ^2,3^ | 2-4 years or more ^6^ |
| **Potential longevity** | 4-6 (10) years ^1^ | > 20 years ^5,6^ |
